# Influencing Primary Care Antibiotic Prescription Behavior Using Financial Incentives

**DOI:** 10.1177/10591478241264022

**Published:** 2024-07-26

**Authors:** Salar Ghamat, Mojtaba Araghi, Lauren E Cipriano, Michael Silverman

**Affiliations:** 1 Lazaridis School of Business and Economics, 8431 Wilfrid Laurier University, Waterloo, ON, Canada; 2 Ivey Business School and the Department of Medicine and the Department of Epidemiology and Biostatistics at Schulich School of Medicine & Dentistry, Western University, London, ON, Canada; 3 Division of Infectious Diseases, Schulich School of Medicine and Dentistry, Western University and London Health Sciences Centre, London, ON, Canada

**Keywords:** Healthcare operations, antibiotic prescription, antibiotic resistance, physician behavior, game theory, incentive payment models

## Abstract

Antibiotic resistance is an ongoing public health crisis fueled by overuse and misuse of antibiotics. The goal of this article is to examine the impact of action-based incentive payments on reducing inappropriate antibiotic prescriptions in primary care, where 30%–50% of antibiotic prescriptions are inappropriate. Various financial incentive programs to reduce the rate of inappropriate antibiotic prescriptions have been implemented and studied empirically. However, there have not been analytical studies to evaluate payment model contract design features and the potential for payment models to impact diagnosis decision making. We develop a stylized physician compensation model to study the interaction between a payer and a provider. The payer offers a payment contract, with a bonus tied to the prescription, to maximize social welfare, considering total costs of providing care and social harm from antibiotic resistance. Given the contract offered and their own opportunity cost associated with factors such as fear of misdiagnosis and time spent explaining to patients why antibiotics are not indicated, the provider chooses whether or not to prescribe antibiotics to patients for whom antibiotics are not clinically indicated. We consider four cases: when diagnostic accuracy relies on symptom presentation versus additional diagnostic testing and when the opportunity cost of not prescribing antibiotics is public versus private information of the provider. When there is no information asymmetry, an action-based incentive payment can co-ordinate care and achieve the first-best policy, decreasing the rate of inappropriate prescribing, even when incentive payments can affect the diagnosis behavior. However, when the diagnosis depends on additional testing, the first-best policy results in fewer inappropriate antibiotic prescriptions, when the test has high specificity. Therefore, when an accurate technical diagnostic is available, a simple to implement action-based incentive payment can be effective in reducing inappropriate antibiotic prescribing. In the realistic setting where the provider’s opportunity cost is private information, an action-based incentive payment cannot eliminate inappropriate antibiotic prescribing. In these settings, the introduction of point of care diagnostics to aid in objective diagnostic criteria will reduce the unintended consequences of the contract.

## 1. Introduction

Antibiotic resistance is an ongoing public health crisis threatening the ability to treat common infectious diseases (WHO, 2021). Antibiotic-resistant infections are associated with prolonged illnesses, worse clinical outcomes, increased healthcare costs, and higher mortality compared to antibiotic-susceptible infections (CDC, 2019). Annually, antibiotic-resistant infections are estimated to be responsible for more than 35,000 deaths in the United States, 33,000 deaths in Europe, and many more in the developing world (Cassini et al., 2019; CDC, 2019).

Antibiotic resistance develops naturally in bacteria through spontaneous mutation and bacterial conjugation (Mazel and Davies, 1999). Very high levels of antibiotic use, including misuse and overuse, creates a selective pressure that increases the prevalence of resistant strains (Spellberg et al., 2008). Global sales of antibiotics for human consumption increased by 39% between 2000 and 2015 with the highest rates of growth in the developing world where previously limited access to antibiotics is improving (Klein et al., 2021). However, even in high-income countries with active stewardship programs, antibiotic consumption continues to rise (Klein et al., 2021). The U.S. Centers for Disease Control and Prevention (CDC) describes an inappropriate antibiotic prescribing the “most important modifiable risk factor for antibiotic resistence” (Sanchez et al., 2016).

Approximately 60% of U.S. antibiotic expenditures for human health are associated with outpatient settings where 30%–50% of antibiotic prescriptions are inappropriate (Sanchez et al., 2016; Silverman et al., 2017). Unnecessary antibiotic prescription rates are particularly high for nonbacterial acute upper respiratory infections (AURIs) presenting to primary care practitioners such as viral bronchitis, rhinosinusitis, and the common cold (Palms et al., 2018). For example, in one study of 185,014 patients diagnosed with nonbacterial AURI during a visit to a primary care practitioner (the most common documented diagnosis was a common cold), 46% of patients were inappropriately prescribed an antibiotic (Silverman et al., 2017). Sore throat represents 3%–6% of all family physician office visits, and, among sore throats in adults, 85%–90% are caused by viral infections where antibiotics are not indicated (Linder and Stafford, 2001; Worrall et al., 2007). Despite the personal harms associated with side effects of antibiotics and the social harm of propagating antibiotic resistance, inappropriate use of antibiotics for the treatment of sore throat is widespread, with antibiotics prescribed in over 70% of visits (Linder and Stafford, 2001).

There are many predictors and potential explanations for inappropriate antibiotic prescribing (reviewed by McKay et al. (2016)). Perceived patient expectations, a lack of accountability related to inappropriate prescribing, and the additional time needed with a patient to explain that antibiotics are not appropriate were identified as the three leading explanations physicians provided for inappropriate antibiotic prescribing to patients they had diagnosed with (viral) acute bronchitis (Dempsey et al., 2014). Observations in pay-per-encounter systems support the hypothesis that pressure to reduce patient visit length and to increase patient throughput increases the frequency of antibiotic over-prescribing (Neprash et al., 2023; Silverman et al., 2017). For example, in a population wide study, primary care physicians seeing less than 25 patients per day were less likely to prescribe antibiotics for nonbacterial upper respiratory infections than were those seeing 25–44 patients/day; those seeing more than 44 patients/day were most likely to over-prescribe (Silverman et al., 2017). Recency of physician training (Degnan et al., 2021), knowledge gap (McCleary et al., 2021), decision fatigue (Linder et al., 2014), a desire to maintain positive relationships with patients who providers perceive to expect or demand antibiotics (Butler et al., 1998; Fletcher-Lartey et al., 2016; Ong et al., 2007), and a fear of misdiagnosis (Sanchez et al., 2014) also contribute to unnecessary prescription of antibiotics. Accounting for practice, physician, and patient features, there remains substantial heterogeneity in rates of inappropriate prescribing across physicians indicating that individual physicians respond to perceived patient expectations, challenges communicating with patients, decision uncertainty, and time pressures influencing inappropriate prescribing differently (Kitano et al., 2021).

Through patient and provider education, barriers to prescribing, standardized infection control practices, and promotion of tests to improve diagnostic accuracy, stewardship programs seek to reduce the inappropriate prescription of antibiotics and extend the useful life of existing antibiotics (reviewed by McDonagh et al., 2018). In addition, financial incentives can be used to influence the antibiotic prescription behavior of physicians (Martens et al., 2007; NHS, 2010). Financial incentives have improved drug prescription practices in many countries including the United States, but variation exists in the effectiveness of these interventions across physician type and by clinical indication (Anyanwu et al., 2020; Ellegård et al., 2018; Kilaru et al., 2021). Analytical studies of financial incentives targeting inappropriate prescribing may help to explain how variation in payment model design features, physician characteristics, and clinical indications may influence their effectiveness in practice.

Our study extends the analysis of incentive payment models to a mechanism seeking to reduce inappropriate antibiotic prescription. In particular, we develop a stylized physician compensation model to study the interaction between a healthcare payer (payer), representing a publicly funded health insurer seeking to maximize health in the population and reduce the social harm of antibiotic resistance, and a care provider (provider) who makes the antibiotic prescription decision for patients presenting for care. Patients present with symptoms corresponding to a probability of having a bacterial infection for which antibiotics are clinically indicated. We assume that only patients with bacterial infection for which antibiotics are clinically indicated benefit from antibiotics; patients with viral infections or bacterial infections for which antibiotics are not clinically indicated do not benefit from antibiotics but do suffer from potential harms associated with adverse drug reactions (Shehab et al., 2008). The payer offers a payment contract to providers to maximize social welfare, defined as the monetary value of health outcomes less the total costs of providing care. Given the contract payment options and their own expected opportunity cost of additional time spent with the patient to explain appropriate non-pharmaceutical treatment and why antibiotics are not indicated, the provider chooses whether or not to prescribe antibiotics to patients diagnosed with an infection for which antibiotics are not clinically indicated. Note that the expected opportunity cost does not need to be restricted to the time spent with the patient; it can include other potential drivers of inappropriate antibiotic prescriptions such as fear of misdiagnosis and the cost of damaging positive relationships with patients expecting antibiotics.

Our article yields several interesting and policy-relevant findings pertinent to fee-for-service (FFS) physician reimbursement systems. We first consider the case when diagnosis is based largely on clinical interpretation of symptom presentation, as occurs for many common conditions presenting to primary care providers. For example, classification of an infection of the middle ear (Segal et al., 2005), infection of the sinuses (Aring and Chan, 2011), or conjunctivitis (commonly called “pink eye”) (Yeu and Hauswirth, 2020) as bacterial or viral is based on the clinical interpretation of symptom presentation. In such cases, diagnostic accuracy relies on a provider’s own clinical interpretation of symptom presentation, meaning that a provider may change their diagnostic criteria in response to the incentive payments. As such, we will discuss how clinically and professionally acceptable diagnoses, within the bounds of what is reasonable, responsible, and ethical for physicians, can limit the unintended impacts of incentive payments. For this scenario, we find that the socially optimal level of antibiotic prescription (first-best policy) is defined by a threshold policy on providers’ diagnosis along the symptom distribution domain. In particular, this threshold is a point along the symptom distribution where the probability of antibiotics being clinically indicated is equal to the ratio of the cost of a false diagnosis of a bacterial infection to the total cost of any false diagnosis. We show that an action-based payment comprised of a fixed visit fee and a bonus payment, paid when no antibiotic is prescribed to a patient for whom it is not clinically appropriate, can co-ordinate the system when the opportunity cost of the provider is public information. A similar action-based payment system in Japan, where medical facilities collect an approximately $7 bonus when physicians do not prescribe antibiotics for outpatients diagnosed with acute upper respiratory infections, demonstrated a 17.8% reduction in antibiotic prescribing (Okubo et al., 2022). Next, we consider the case when a provider uses technical investigations to inform diagnosis (e.g., rapid antigen detection or throat culture for the evaluation of group A streptococcus infection in patients presenting with a sore throat (Cohen et al., 2016)). In this case, we find that the socially optimal level of antibiotic prescription is defined by a threshold policy along the symptom distribution such that the provider prescribes antibiotics to patients diagnosed with viral infection only if their symptoms are higher than a threshold. We show that adding an action-based bonus payment can coordinate antibiotic prescription decisions to socially optimal levels. Importantly, while action-based bonus payments can coordinate antibiotic prescription regardless of the diagnostic method, we demonstrate that the use of technical diagnostic testing with high specificity can lead to a reduction in inappropriate antibiotic prescriptions compared to cases where the diagnosis relies solely on a physician’s interpretation of symptoms.

In the realistic case when the opportunity cost is private information, financial incentive imposes a moral hazard on the provider’s prescription decision, increasing the likelihood of inappropriate antibiotic prescription to a patient with viral infection. Shifting the physician’s prescription characteristics reduces the effectiveness of coordinating contracts and the first-best antibiotic prescription policy is no longer achievable. In this case, incentive payments still reduce the rate of inappropriate prescriptions; however, providers with relatively higher opportunity cost continue to over-prescribe compared to the first-best policy and providers with relatively lower opportunity cost may shift their prescription criteria in order to increase the frequency with which they earn the bonus. Through extensive numerical studies, we show that a menu of contracts does not significantly improve the outcome achieved with a single contract.

Finally, we apply our framework to patients presenting to primary care providers with a sore throat. While 85%–90% of sore throats are caused by viral infections, the most common bacterial cause of sore throat is group A β -hemolytic streptococci (GABHS or “step throat”) (Linder and Stafford, 2001; Worrall et al., 2007). Left untreated, GABHS infection can result in serious complications. For patients with GABHS infection, antibiotics provide symptom relief as well as reduce the risk of throat abscess, acute rheumatic fever, and poststreptococcal kidney disease (Choby, 2009; Kalra et al., 2016). Antibiotics used to treat GABHS have common side effects including rash, hives, diarrhea, among other symptoms (Gillies et al., 2015; Soukavong et al., 2016). We apply our model to demonstrate that a bonus payment may influence physician diagnostic behavior at the expense of health outcomes in patients with bacterial infections when diagnostic accuracy relies on symptom presentation alone. We then demonstrate the benefit of a bonus to reduce antibiotic prescribing behavior when diagnostic accuracy relies on a point-of-care rapid antigen test, and calculate the optimal bonus payment for not prescribing antibiotics to patients diagnosed with viral infections causing sore throat. We present results of this case study in Section 6; detailed exposition of all assumptions and inputs are presented in the E-companion.

## 2. Literature Review

### 2.1. Programs to Reduce Antibiotic Prescribing Behavior

The use, even appropriate use, of antibiotics creates a selection pressure on bacterial populations increasing the prevalence of antibiotic resistant strains such that, ultimately, antibiotics are a limited resource. High levels of antibiotic resistance affect the ability to treat common pathogens including *S. aureus*, *S. pneumoniae*, *E. coli*, group A *Streptococcus*, and *C. difficile* (CDC, 2019; Spellberg et al., 2008; WHO, 2021). However, individual patients, doctors, and antibiotic manufacturers have little incentive to moderate consumption. Numerous antibiotic stewardship programs have been implemented to limit the overuse and misuse of antibiotics (Davey et al., 2013; Johnson et al., 2015; McDonagh et al., 2018). For example, some programs implement antibiotic oversight committees to enforce clinical guidelines (e.g., Nathwani et al., 2011), restrict prescribing (e.g., Wang et al., 2020), aim to improve communication between patients and clinicians (e.g., Cals et al., 2013), inform clinicians about their own prescribing rates relative to their peers (e.g., Milani et al., 2019), leverage the value of public commitments to the judicious use of antibiotics (e.g., Meeker et al., 2014), and provide clinical education for healthcare professionals (e.g., Weiss et al., 2011). Multifaceted strategies have been shown to be most effective at reducing inappropriate antibiotic prescriptions (Llor and Bjerrum, 2014).

Empirical studies evaluating financial incentive programs have shown reductions in overall antibiotic prescribing and, in some cases, specific reductions in inappropriate prescribing. Yip et al. (2014) find that salary capitation combined with a pay-for-performance (P4P) payment model decreased antibiotic prescription by 15% in rural northwest China. Gong et al. (2016) showed that a regular review of prescription practices, feedback, and financial penalty for inappropriate prescribing dramatically reduced total inpatient and outpatient prescriptions. Financial incentives introduced to target inappropriate antibiotic prescribing by primary care physicians in the United Kingdom had the greatest impact on practices with a high baseline rate of prescribing (Anyanwu et al., 2020). Martens et al. (2007) showed that a one-time financial incentive that is independent of behavior leads to a small and short term change in prescribing practices. Ellegård et al. (2018) found that, in Sweden, a P4P model focused on shifting prescription patterns towards narrow-spectrum antibiotics (vs. broad-spectrum antibiotics) significantly increased the proportion of prescriptions that were narrow-spectrum. In contrast to these empirical studies, in this article, we design co-ordinating contracts that, in addition to considering healthcare spending, also attempt to improve the health outcome of patients by reducing the inappropriate use of antibiotics.

### 2.2. Coordination Mechanisms in Healthcare and Supply Chain

Our research is connected to the stream of literature in healthcare operations and supply chain management that uses applied game-theory to design coordination mechanisms (Betcheva et al., 2021; Keskinocak and Savva, 2020; Tsay and Agrawal, 2004). These studies mainly focus on outcome or performance-based coordination mechanisms. A stream of healthcare operations management studies focuses on payment contracts between payers and care providers, such as gainsharing agreements between a hospital and physicians (Gupta et al., 2021), bonus compensation for competitive providers (Jiang et al., 2020), and reference pricing (Nassiri et al., 2022). Bravo et al. (2023) and Ghamat et al. (2021) proposed performance-based payments to incentivize care coordination between post-acute care providers and hospitals, encouraging cost reduction and high-quality care as required by Medicare’s bundled payment models. Unlike these studies, we investigate an *action-based* payment model, where contract payments depend on the treatment choice of the physician. This distinct modeling feature captures a unique challenge of applying incentive payments in the primary care setting: the payer cannot evaluate provider decisions by monitoring the health outcome of all patients seeking care for upper respiratory symptoms.

Another stream of work evaluates payment models to incentivize providers to adopt the optimal treatment choice or intensity. Yaesoubi and Roberts (2011) studied payment models that achieve the optimal use of cancer screening tests when the provider is strictly concerned about the cost of treatment. Dai et al. (2017) evaluated the effect of a provider’s misdiagnosis concerns and a reimbursement ceiling on diagnostic test-ordering behavior. Ghamat et al. (2018) evaluated contracts that incentivize providers to use a genetic test for optimal chemotherapy prescription. Similar to our approach to modeling the provider’s altruistic behavior when patients receive inappropriate treatment, Dai et al. (2017) and Ghamat et al. (2018) explicitly modeled the provider’s concern about misdiagnosis and show that both over-testing and under-testing are possible outcomes of these concerns. Andritsos and Tang (2018) considered a co-managed situation where both the provider and the patient can exert effort to reduce hospital readmission rates. They show that bundled payment outperforms FFS by causing the provider and the patient to exert more effort to reduce readmission. Adida et al. (2017) study the impact FFS and bundled payment models have on patient selection and treatment intensity decisions finding that FFS results in over-treatment of patients. In another study, Adida and Dai (2023) examined the impact of physician compensation on their diagnostic effort and use of confirmatory tests. Unlike our study, these studies do not incorporate the misuse of treatment and the social harm of over-testing/over-treatment. Moreover, in addition to studying the impact of payment models on treatment choice, we evaluate potential unintended consequences of incentive payment models on the diagnosis behavior of a provider.

## 3. Model

### 3.1. Overview

We consider a system consisting of a payer, a healthcare provider, and a sequence of patients experiencing symptoms which could be due to either a bacterial infection for which antibiotics are clinically indicated (“bacterial”) or an infection (viral or bacterial) for which antibiotics are not clinically indicated (denoted for succinctness in the analysis sections as “viral”). We explore the existence and features of payer–provider contracts designed to reduce antibiotic prescriptions for viral infections and maximize social welfare. Because it is not possible for the payer to observe the true health state of all patients prescribed antibiotics nor to observe the social harm of increased antibiotic resistance from each inappropriate prescription, we focus on alignment of diagnosis and prescription decisions. We investigate a simple action-based payment contract in which the payer offers a contract $\zeta =\{w,b\}\in R^{2}$ for patient-care services, where w represents the per-visit fee and b represents a bonus paid only when the provider does not prescribe antibiotics to a patient they have diagnosed with a viral infection. Similar payment models have been implemented in healthcare systems to influence drug prescription behaviours (reviewed by Rashidian et al. 2015). For example, beginning in 2018, nearly 3000 medical facilities in Japan could claim a small financial reward, approximately US$7, when physicians did not prescribe antibiotics for outpatients with either acute upper respiratory infections or acute gastroenteritis (Okubo et al., 2022). In the common fee-for-service payment structure for primary care practitioners, the bonus payment for not prescribing antibiotics for a patient diagnosed with a viral infection can be implemented as an add-on payment for billing purposes, similar to the approach used to bill for point-of-care diagnostics and smoking cessation intervention (Ontario Ministry of Health and Long Term Care, 2023).

Our mathematical model captures trade-offs faced by physicians. Physicians are motivated to diagnose patients correctly and not to prescribe antibiotics inappropriately through an altruistic utility they obtain from a positive change in their patients’ health. However, factors such as fear of misdiagnosis, challenges communicating with patients, desire to maintain positive relationships with patients, and the additional time it can take to explain why antibiotics are not indicated and to recommend a course of non-pharmaceutical treatment when patients are expecting an antibiotic imposes an opportunity cost that incentivizes physicians to prescribe antibiotics to patients diagnosed with viral infection (Butler et al., 1998; Dempsey et al., 2014; Silverman et al., 2017).

### 3.2. Patient Encounter and Patient Outcomes

Consider a sequence of patient–provider encounters where, at each encounter, a patient presents with symptoms that are denoted by a random variable s∈[0,1] with density function $f_{s}(.)$ . Each symptom profile s captures physical symptoms, age, prior health (e.g., risk–factors for type of infection), and time of year or other similar environmental information, and thus corresponds to a probability of bacterial infection p(s) , and of viral infection 1−p(s) . Without loss of generality, we assume symptom profiles are arbitrarily sorted based on the increasing likelihood of bacterial infection allowing a semi-ordering of symptoms based on the relative likelihood of each type of infection; in other words, p(s) is increasing in s .

We present health benefits, which naturally occur in terms of alleviated symptoms and severe outcomes averted but may also include monetary gains from averting lost days of paid work, solely into monetary units. In practice this unit-conversion can be done using the marginal willingness-to-pay threshold of the decision maker for health benefits (Neumann et al., 2016). Treatment with antibiotics provides an expected monetary health benefit B>0 for patients with bacterial infection. Patients who receive antibiotics may also experience antibiotic–associated adverse events such as diarrhea, dizziness, and headache. The expected cost of side effects from antibiotic treatment is denoted by F≥0 . We assume that B>>F , therefore, patients with a bacterial infection receive higher expected utility from antibiotics as opposed to not receiving them.

The provider may also prescribe antibiotics for patients diagnosed with viral infection because of uncertainty in the diagnosis and/or the opportunity cost of the provider rising from factors such as challenges communicating with patients, desire to maintain positive relationships with patients, and time spent explaining to a patient who expects antibiotics why they are not indicated. Therefore, the provider makes a decision d(s)∈{0,1} about whether to prescribe antibiotics to a patient diagnosed with a viral infection with symptom s .

Since the likelihood of a bacterial infection increases with s , the chance of a false viral (FV) diagnosis is higher for patients exhibiting more bacterial-type symptoms (i.e., with larger s ). These patients also have greater anticipation of antibiotics; thus, the provider will need to spend additional time with these patients providing an explanation about why antibiotics are not prescribed. Therefore, as s increases, the provider’s incentive to prescribe antibiotics to patients diagnosed with a viral infection changes monotonically. As a result, the provider’s optimal prescription decision follows a threshold policy, as shown in Lemma 1.

Lemma 1 The provider’s decision d(s)∈{0,1} on whether to prescribe antibiotics to a patient, with symptom s , diagnosed with a viral infection follows a threshold policy.

Lemma 1 suggests that under the optimal prescription policy, the provider prescribe antibiotics to a patient diagnosed with a viral infection if and only if they exhibit symptoms more consistent with a bacterial infection over a specific threshold in s , denoted by τ . Following such policy, the provider will save their opportunity cost and, at the same time, reduces the negative effect of the uncertainty in the diagnosis.

Remark 1 Factors beyond patients’ symptoms (e.g., communication challenges, understanding of viruses compared to bacteria, or their relationship with the provider) may also affect the opportunity cost of the provider. Our model assumes that these personal characteristics are evenly distributed along the symptom levels, so they do not affect the provider’s prescription decision on threshold τ .

**Figure 1.**
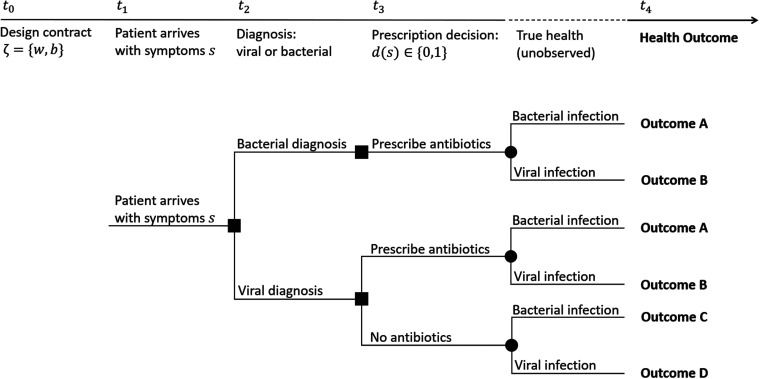
Sequence of events. The provider diagnoses the patient as having a bacterial or viral infection and then decides d(s) , whether to prescribe antibiotics to the patient if they are diagnosed with a viral infection.

A schematic of sequence of events leading to possible patient health outcomes, with their corresponding probabilities for symptom profile s , is presented in Figure 1.

Patients who truly have bacterial infection and receive antibiotics (Outcome A) receive health benefit B and incur side effect F . We represent the change in their health by (B,F) , which can occur either because they are diagnosed correctly with a bacterial infection, or the provider decides to prescribe them antibiotics after (incorrectly) diagnosing them with a viral infection. Patients with a viral infection may also receive antibiotics either because they are misdiagnosed with a bacterial infection or because the provider decides to prescribe antibiotics despite a viral infection diagnosis (Outcome B). These patients do not obtain any health benefit from antibiotics but may experience some antibiotic–associated side effects, thus their health changes by (0,F) . Patients with a bacterial infection who are initially misdiagnosed with viral infection and are not prescribed antibiotics will revisit the provider when their health worsens and will be prescribed antibiotics at that time. Prolonged bacterial infection and delayed care extends the period of illness and can reduce the health benefit from using antibiotics (Spurling et al., 2017). We capture the harm of delayed care with a discount factor 0<δ<1 , ultimately yielding the change (δB,F) in the patient’s health (Outcome C). Finally, a patient with viral infection who does not receive antibiotics will recover naturally and will not experience benefits or side effects associated with antibiotics (Outcome D). Thus, the change in their health will be (0,0) . We define $P(s,\tau )=\{P_{A}(s,\tau ),P_{B}(s,\tau ),P_{C}(s,\tau ),P_{D}(s,\tau )\}$ as the set of probabilities associated with each health outcome for a patient at symptom level s , which enables us to define the provider’s and the payer’s decision problems in next sections.

### 3.3. The Provider’s Decision Problem

Providers obtain three types of rewards. First, we assume that the provider is altruistic, in that they care about the health outcome of their patients, and receives a utility of $U_{P}(\cdot ,\cdot )\in R$ from change (⋅,⋅) in a patient’s health, where $U_{P}(B,F)>U_{P}(\delta B,F)>U_{P}(0,0)>U_{P}(0,F)$ . A similar modeling approach has been widely used in the literature to incorporate the provider’s concerns regarding the health outcome of their patients (e.g., Adida et al., 2017; Ghamat et al., 2018). Second, the provider receives payments $\zeta =\{w,b\}\in R^{2}$ from the payer in exchange for providing patient care. Finally, the provider faces an opportunity cost k(s)≥0 which captures the monetary value associated with the provider’s incremental visit time spent explaining to a patient who expects antibiotics why antibiotics are not an appropriate treatment. We assume that k(s) is non-decreasing in s , as, intuitively, patients’ expectations of receiving antibiotics would increase with the alignment of their symptoms with bacterial infection. Providers are all paid the same rate, therefore, in the main model we assume that providers are homogeneous in their opportunity cost. However, because of differences in physician response to the perceived expectation of patients for antibiotics and challenges communicating with patients due to patient age, patient education, provider recency of training, provider confidence in their diagnosis, and quality of the patient–provider relationship (Dempsey et al., 2014; Kitano et al., 2021), there may be heterogeneity in the expected opportunity cost of providers, which we will address as a model extension in Section 7.

Given the contract ζ={w,b} offered by the payer, the providers make two decisions. First, they diagnose the patient with either a bacterial or viral infection, based on the clinical interpretation of symptoms or through a technical diagnostic test. We discuss the former in Section 4 and the latter in Section 5. If the patient is diagnosed with bacterial infection, the provider prescribes antibiotics. In cases of a viral diagnosis, the provider decides which patients should receive antibiotics (i.e., chooses threshold τ ), in order to maximize their expected payoff function, defined as follows:

(1) $$\begin{array}{rl} & \Pi_{P}(\tau ,B,F,k,\zeta )\\ & \;=\int_{0}^{1}\left(\begin{array}{rl} & P_{A}(s,\tau )\left[U_{P}(B,F)+w\right]\\ & \;+\;P_{B}(s,\tau )[U_{P}(0,F)+w]\\ & \;+\;P_{C}(s,\tau )[U_{P}(\delta B,F)+2w+b-k(s)]\\ & \;+\;P_{D}(s,\tau )[U_{P}(0,0)+w+b-k(s)] \end{array}\right)f_{s}(s)\;ds, \end{array}$$

where each term corresponds to the provider’s monetary benefit associated with each possible patient health outcome. In addition to utility of $U_{P}(\cdot ,\cdot )$ and the visit fee w , in Outcomes C and D the provider reward includes earning bonus b and incurring opportunity cost k(s) in the visit when antibiotics were not prescribed.

Providers (and payers) do not observe the true health state of the patient. Because outcomes are probabilistic at the individual level, a patient who feels better after a few days may (falsely) attribute this to treatment with an antibiotic and may attribute adverse events from the antibiotic to the original illness. An outcome-based payment is therefore not practical or feasible which is why we propose a fully observable action-based payment where the payer receives bonus b for the action combination of a viral diagnosis and no antibiotic prescription (Outcomes C and D). Moreover, we assume in Outcome C, the provider receives a second visit fee when the patient returns with persistent or progressive symptoms consistent with normal practice in fee-for-service primary care. Note that, for family practitioners it is typical to receive a second visit fee for a follow-up visit; however, in hospital settings, re-admissions are often coded as a lower charge. Nevertheless, our results are robust even when there is no bonus payment for misdiagnosed bacterial infections or second visit fee (details of such analysis are presented in the E-companion).

### 3.4. The Payer’s Decision Problem

We assume that the payer’s objective is to maximize social welfare, which is the monetary value of patient health outcomes minus the cost of providing care and the social harm of prescribing antibiotics (increased levels of antibiotic resistance). The payer obtains a reward $U_{S}(\cdot ,\cdot )\in R$ generated by the health outcome of a patient. We assume that the payer’s utility function is more responsive to patient health than the provider’s utility function, that is, $U_{P}(\cdot ,\cdot )=\alpha U_{S}(\cdot ,\cdot )$ , where α∈(0,1) represents the provider’s altruism. The payer incurs the cost of antibiotics, c≥0 , prescribed by the provider. The payer also incurs the expected value of the social harm of antibiotic resistance associated with inappropriate and unnecessary antibiotic prescription, which we denote by h≥0 . Even though appropriate antibiotic use also contributes to the selection pressure that perpetuates antibiotic resistance, in our model, the use of antibiotics for bacterial infections is inevitable and so we limit the accounting of social harm to the case of an inappropriate antibiotic use only (Outcome B). Thus, the payer’s expected payoff function takes the form of

(2) $$\begin{array}{rl} & \Pi_{S}(\tau ,B,F,c,h,\zeta )\\ & \;=\int_{0}^{1}\left(\begin{array}{rl} & P_{A}(s,\tau )\left[U_{S}(B,F)-w-c\right]\\ & \;+P_{B}(s,\tau )[U_{S}(0,F)-w-c-h]\\ & \;+P_{C}(s,\tau )[U_{S}(\delta B,F)-2w-b-c]\\ & \;+P_{D}(s,\tau )[U_{S}(0,0)-w-b] \end{array}\right)f_{s}(s)\;ds. \end{array}$$

The payer seeks to identify contract terms that will maximize their expected payoff function subject to the behavior of the provider.

Remark 2 A contract structure where the payer penalizes the provider for prescribing antibiotics to a patient they have diagnosed with a viral infection leads to a parallel mechanism design and the same optimal solutions. This is because when deciding on antibiotic prescription for patients with viral diagnosis, the provider compares her profit from prescribing antibiotics (minus any applicable penalty) with her profit from not prescribing antibiotics (plus any applicable bonus). Therefore, the penalty and bonus can be captured with a single incentive payment either for prescribing or not prescribing antibiotics.

## 4. Optimal Contract When Diagnosis Relies on Symptom Presentation

When diagnosis is established by clinical interpretation of symptom presentation, providers may vary in their diagnostic accuracy depending on how closely they adhere to clinical guidelines, variation in patient physiology and ability to describe symptoms, and potential confounding or masking of symptoms due to concomitant conditions or their treatment. In this situation, it is possible that bonus payments may not only affect physician prescribing decisions, but also unintentionally affect diagnosis decisions. If this occurs, it could harm the health of patients with bacterial infections, who would have, under current reimbursement policy, received a correct diagnosis of bacterial infection and, appropriately, treatment with an antibiotic. If physicians shift their diagnosis criteria in an effort to secure the bonus payment more often, patients with bacterial infections may incorrectly be diagnosed as viral infections delaying correct diagnosis and appropriate treatment.

**Figure 2.**
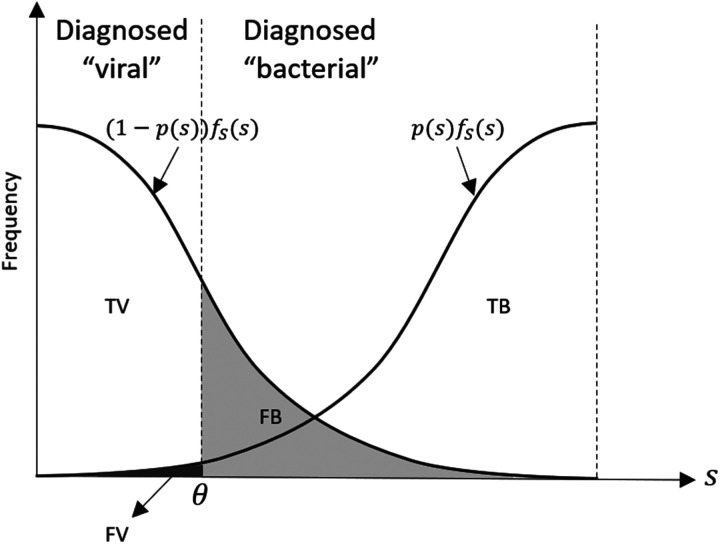
Illustration of the possible diagnostic outcomes over the range of presenting symptoms, using normal distribution for patient symptoms and p(s)=s .

Since we assume that symptom profiles are arbitrarily sorted based on the increasing likelihood of bacterial infection (i.e., p(s) is increasing in s ), the provider may apply a threshold policy to differentiate viral and bacterial infections. We define θ as the threshold along the symptom domain whereby the provider diagnoses a patient with symptoms above the threshold as having a bacterial infection (and otherwise diagnoses the patient as having a viral infection). For analytical simplicity, we initially set the domain of θ to be the entire range of possible symptom presentations, that is, θ∈[0,1] ; we then discuss the case where θ is limited within the domain of s by professionally acceptable bounds. Therefore, the symptom threshold diagnosis policy will result in four possible outcomes, as shown in Figure 2: (1) true viral (TV): patients correctly diagnosed with a viral infection (i.e., $\int_{0}^{\theta }(1-p(s))f_{s}(s)\;ds$ ); (2) true bacterial (TB): patients correctly diagnosed with a bacterial infection (i.e., $\int_{\theta }^{1}p(s)f_{s}(s)\;ds$ ); (3) false viral (FV): patients with bacterial infection that are misdiagnosed as having viral infection (i.e., $\int_{0}^{\theta }p(s)f_{s}(s)\;ds$ ); and (4) false bacterial (FB): patients with viral infection that are misdiagnosed as having bacterial infection (i.e., $\int_{\theta }^{1}(1-p(s))f_{s}(s)\;ds$ ).

After diagnosing patients with either a viral or bacterial infection, the provider chooses the prescription threshold τ for those diagnosed with a viral infection, that is, τ≤θ . We can then combine the diagnosis and prescription decisions into the health outcome groups identified in Figure 1 and construct the probability of each health outcome as follows:

(3) $$\begin{array}{rl} P_{A}(s,\tau ,\theta ) & =I_{\theta <s}\;p(s)+I_{\tau <s\leq \theta }\;p(s),\\ P_{B}(s,\tau ,\theta ) & =I_{\theta <s}\;\left(1-p(s)\right)+I_{\tau <s\leq \theta }\;\left(1-p(s)\right),\\ P_{C}(s,\tau ,\theta ) & =I_{s\leq \tau }\;p(s),\\ P_{D}(s,\tau ,\theta ) & =I_{s\leq \tau }\;\left(1-p(s)\right). \end{array}$$

where I is an indicator variable that takes on the value 1 when a specified condition is true and 0 when the condition is false. Incorporating the provider’s decision over the diagnostic threshold, θ , as well as the prescription threshold, τ , when diagnosis relies on interpretation of symptom presentation, the payer’s problem is as follows:

(OPT1) $$\begin{array}{rl} & \max_{\zeta \in R^{2}}\;\Pi_{S}(P\left(\tau *,\theta *\right),B,F,c,h,\zeta )\\ \text{s.t.} & \{\tau *,\theta *\}=\underset{0\leq \tau \leq \theta \leq 1}{\arg\;\max}\;\Pi_{P}(P\left(\tau ,\theta \right),B,F,k,\zeta )\\ & \Pi_{P}(P\left(\tau *,\theta *\right),B,F,k,\zeta )\geq {\underline{\Pi }}_{P}. \end{array}$$

The first set of constraints are the incentive constraints such that, under the payment contract, the provider’s optimal diagnosis and treatment thresholds are $\theta^{*}$ and τ* , respectively. The second set of constraints are the provider’s participation (individual-rationality) constraints to ensure that the contract offered by the payer results in higher payoff for the provider compared to their status quo payoff (denoted by ${\underline{\Pi }}_{P}$ ).

In this optimization problem, the health outcome of a patient depends on two interacting decisions by the provider: the diagnostic threshold θ∈[0,1] and the antibiotics prescription threshold τ≤θ . However, it follows from equation (3) that the probability of each health outcome (and thus the provider’s payoff) can be written independent of the diagnostic threshold θ , that is, we can write the probability of Outcome A as $P_{A}(s,\tau ,\theta )=I_{\tau <s}p(s)$ and Outcome B as $P_{B}(s,\tau ,\theta )=I_{\tau <s}(1-p(s))$ . Therefore, for the purpose of studying changes in the prescription behavior of providers, it is mathematically sufficient to simplify the decision space such that the diagnosis and prescribing behaviour are always aligned (i.e., the provider sets θ*=τ* ) and then identify the optimal prescription threshold.

Remark 3 In practice, θ may be constrained by professionally acceptable bounds such that $0<\underline{\theta }\leq \theta \leq \bar{\theta }<1$ . In this case, if setting θ*=τ* in the optimization problem (OPT1) yields $\theta^{*}<\underline{\theta }$ , then the diagnostic threshold should be set at $\underline{\theta }$ . However, the total proportion of patients prescribed antibiotics, the sum of those diagnosed with a bacterial infection (i.e., $\int_{\underline{\theta }}^{1}f_{s}(s)ds$ ) and those diagnosed with a viral infection and prescribed antibiotics (i.e., $\int_{\tau^{*}}^{\underline{\theta }}f_{s}(s)ds)$ , will be equivalent to the proportion diagnosed with a bacterial infection (and so prescribed antibiotics) in the case where θ is not restricted. Because the proportion of patients receiving antibiotics is consistent in both situations, without loss of generality, we assume that θ∈[0,1] throughout the rest of the article.

We first analyse optimal antibiotic prescription behavior in an integrated care system as a benchmark (Section 4.1) and then we investigate if an action-based payment model can coordinate care and achieve antibiotic prescription levels similar to the integrated care system (Section 4.2).

### 4.1. Integrated Care System

To identify the socially optimal use of antibiotics, we define an integrated care system such that the central planner’s objective function is the sum of the payer and the provider’s payoff functions. We define the socially optimal level of antibiotic prescription as the first-best antibiotic prescription policy. Note that in the integrated care system there is no monetary transfer between the two parties. The central planner chooses the prescription (and thus the diagnosis) threshold τ∈[0,1] in order to maximize their expected payoff function:

(4) $$\begin{array}{rl} & \Pi_{C}(\tau ,B,F,c,h,k)=\int_{0}^{1}\\ & \;\left(\begin{array}{rl} & P_{A}(s,\tau )\left[U_{S}(B,F)+U_{P}(B,F)-c\right]\\ & \;+P_{B}(s,\tau )[U_{S}(0,F)+U_{P}(0,F)-c-h]\\ & \;+P_{C}(s,\tau )[U_{S}(\delta B,F)\\ & \;+U_{P}(\delta B,F)-c-k(s)]\\ & \;+P_{D}(s,\tau )[U_{S}(0,0)+U_{P}(0,0)-k(s)] \end{array}\right)f_{s}(s)\;ds. \end{array}$$

**Figure 3.**
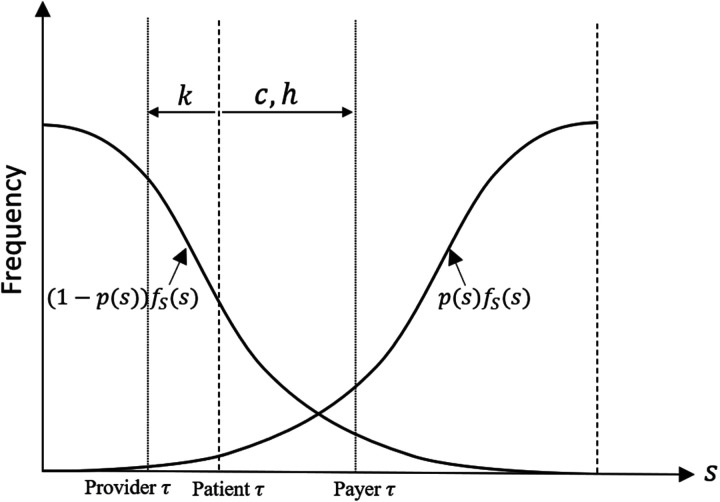
Impact of the provider’s opportunity cost ( k ), the cost of antibiotics ( c ), and the social harm of antibiotic resistance ( h ) on the prescription threshold preferences from different perspectives.

Figure 3 illustrates how the central planner’s prescription threshold is affected by the trade-off between the provider and the payer preferences. The provider’s opportunity cost incentivizes them towards more antibiotic prescriptions and so more bacterial diagnoses (smaller τ ) than is optimal from the patient’s perspective, which only takes into account the health benefits of antibiotics (i.e., B and F ). Conversely, the cost of antibiotics and the social harm of antibiotic resistance lead the payer to prefer fewer antibiotic prescriptions (larger τ ) compared to patients’ expectations. A low value for τ increases the fraction of patients with a viral infection who are misdiagnosed and receive antibiotics. A high value for τ increases the fraction of patients with bacterial infections who will not be diagnosed accurately at their first visit and will need to return when their symptoms worsen.

Therefore, when choosing the prescription threshold τ , the central planner is at risk for two types of prescribing error: prescribing antibiotics to patients with a viral infection (over-prescribing), and not prescribing antibiotics to patients with a bacterial infection (under-prescribing). Over-prescribing causes the central planner to incur the cost of prescribing antibiotics for patient with a viral infection (Outcome B), which is (1+α)F+c+h , instead of the cost of not prescribing (Outcome D), which is k(τ) . Therefore, the cost of over-prescribing is (1+α)F+c+h−k(τ) . Similarly, the central planner’s cost of under-prescribing is the difference between the cost of not-prescribing (Outcome C) and prescribing (Outcome A) antibiotics to a patient with a bacterial infection, that is, k(τ)+(1+α)B(1−δ) .

Because the true health status of a patient is unknown, the diagnosis and prescription decisions are made given symptom presentation of a patient. Therefore, the central planner should diagnose a patient with symptom level τ as viral and not prescribe antibiotics if the expected gain from avoiding over-prescribing (i.e., if the patient is a TV which occurs with probability 1−p(τ) ), exceeds the expected loss of under-prescribing (i.e., if the patient is a FV, which occurs with probability p(τ) ). As a result, the central planner will not prescribe antibiotics to a patient with symptom level τ if and only if $k(\tau )\leq {\hat{k}}^{\text{ P}}(\tau )$ where

(5) $$\begin{array}{r} {\hat{k}}^{\text{ P}}(\tau )=(1-p(\tau ))((1+\alpha )F+c+h)-p(\tau )((1+\alpha )B(1-\delta )). \end{array}$$

Parameter ${\hat{k}}^{\text{ P}}(\tau )$ can be interpreted as the provider’s opportunity cost threshold for patients at symptom level τ . Note that an antibiotic prescription to a patient with symptom level τ does not depend on the symptom distribution $f_{s}(.)$ . This is because the probability of a patient having bacterial infection at a certain symptom level does not depend on the frequency of that symptom level.

Since the provider’s opportunity cost threshold ${\hat{k}}^{\text{ P}}(\tau )$ is decreasing and the opportunity cost k(τ) is increasing in τ , the optimal threshold τ* satisfies ${\hat{k}}^{\text{ P}}(\tau *)=k(\tau )$ as presented in Proposition 1.

Proposition 1 When diagnosis relies on clinical interpretation of symptom presentation, in an integrated care system,

1. if $k(0)>{\hat{k}}^{\text{ P}}(0)$ , the provider prescribes antibiotics to all patients (i.e., τ*=0 ),
2. if $k(0)\leq {\hat{k}}^{\text{ P}}(0)$ , the provider prescribes antibiotics if and only if a patient’s symptom level is above the symptom threshold τ* where
τ*=arg{(1+α)F+c+h−k(τ)(1+α)F+c+h+(1+α)B(1−δ)}0≤τ≤1:p(τ)=(1+α)F+c+h−k(τ)(1+α)F+c+h+(1+α)B(1−δ)}.

As can be seen in Figure 2, shifting the prescription threshold to the right decreases the chance of FB (decreasing the risk of over-prescribing) and increases the chance of FV (increasing the risk of under-prescribing). Therefore, following the same logic as the critical fractile in the classic Newsvendor problem (Silver et al., 1998), at the optimal threshold τ* , the chance of a bacterial infection is equal to the ratio of the cost of over-prescribing to the total cost of over- and under-prescribing (Proposition 1.2). Moreover, as the provider’s opportunity cost increases, prescribing antibiotics becomes increasingly desirable (i.e., τ* decreases in k(s) ), until it reaches the improbable case of prescribing antibiotics to all patients (Proposition 1.1).

Remark 4 To focus on the realistic cases, throughout the article, we present our propositions assuming that our parameter values (i.e., the prevalence of the bacterial infection, sensitivity and specificity of the diagnosis, and the cost of over- and under-prescribing) guarantee the optimality of prescribing antibiotics to patients diagnosed with bacterial infection, regardless of the opportunity cost of the provider (i.e., ${\hat{k}}^{\text{ P}}(1)<0$ ). However, this is not a restrictive assumption, and proofs in E-companion are provided for the general case.

### 4.2. Decentralized Care System

**Figure 4.**
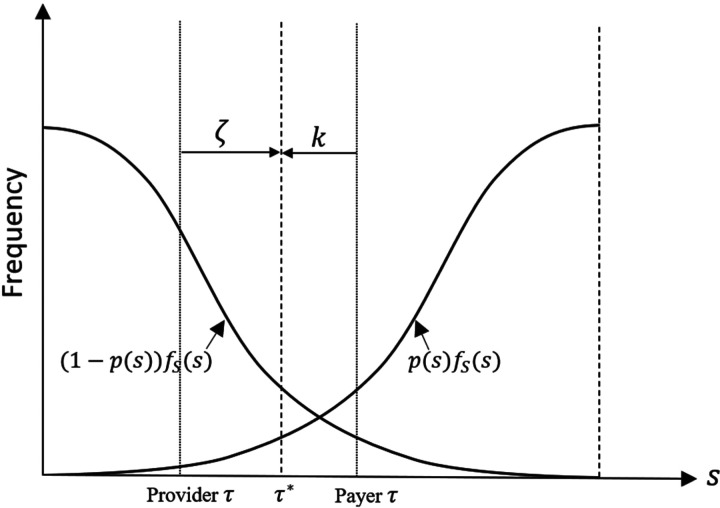
Impact of payment contract ( ζ ) and opportunity cost ( k ) on the optimal prescription threshold τ* .

In this section, we study the decentralized system where the provider’s antibiotic prescription behavior depends on the contract payment terms offered by the payer. Figure 4 illustrates how the forces of the payer’s contract offer balance the provider’s opportunity cost, leading to the selection of a prescription threshold at the socially optimal level (i.e., τ* ). We first present the provider’s optimal antibiotic prescription behavior in response to the contract payment terms offered by the payer. Then, we discuss the payer’s problem and the optimal contract payment terms.

Similar to case of the central planner, diagnosing a patient with symptom level τ with viral infection and not prescribing antibiotics avoids over-prescribing with probability 1−p(τ) , and risks under-prescribing with probability p(τ) . The cost of over- and under-prescribing for the provider is αF−k(τ)+b and −b+αB(1−δ)+k(τ)−w , respectively. Thus, the provider’s response to a payment contract ζ={w,b} is to not prescribe antibiotics to a patient with symptom level τ or lower if the expected gain from avoiding over-prescribing is higher than the expected loss of under-prescribing. Specifically, the provider does not prescribe antibiotics to patients when $b\geq {\underline{b}}^{\text{ P}}(\tau )$ , where

(6) $$\begin{array}{r} {\underline{b}}^{\text{ P}}(\tau )=-(1-p(\tau ))(\alpha F-k(\tau ))+p(\tau )(\alpha B\left(1-\delta \right)+k(\tau )-w). \end{array}$$

For any given visit fee w , ${\underline{b}}^{\text{ P}}(\tau )$ is the minimum bonus required for the provider to not prescribe antibiotics to a patient with symptom level τ or lower. Since ${\underline{b}}^{\text{ P}}(\tau )$ is increasing in τ , the provider chooses threshold τ(w,b) such that ${\underline{b}}^{\text{ P}}(\tau (w,b))=b$ , which yields Lemma 2.

Lemma 2 When diagnosis relies on clinical interpretation of symptom presentation, given a payment contract ζ={w,b} , the provider prescribes antibiotics if and only if a patient’s symptom level is above the threshold $\tau^{*}\left(w,b\right)$ where

(7) τ*(w,b)={0if b<b_ P(0);arg{=αF−k(τ)+bαF+αB(1−δ)−w}0≤τ≤1:p(τ)=αF−k(τ)+bαF+αB(1−δ)−w}if b_ P(0)≤b≤b_ P(1);1if b>b_ P(1).

Lemma 2 shows that the provider sets the optimal prescription threshold τ* such that for a patient with symptom level τ* the chance of a bacterial infection is equal to the ratio of the cost of over-prescribing to the total cost of over- and under-prescribing (i.e., the critical fractile). If $b<{\underline{b}}^{\text{ P}}(0)$ , the cost of under-prescribing or the prevalence of the bacterial infection is very high, and the provider prescribes antibiotics to all patients ( τ*(w,b)=0 ). If $b>{\underline{b}}^{\text{ P}}(1)$ , the cost of under-prescribing or the prevalence of the bacterial infection is very low, and the provider does not prescribe antibiotics to any patient ( τ*(w,b)=1 ).

Finally, the payer is also looking to balance their cost of over- and under-prescribing, which are F+c+h−b and b+B(1−δ)+w , respectively. Knowing the provider’s response, the payer offers the minimum bonus required by the provider to not prescribe antibiotics to a patient with symptom level τ , that is, ${\underline{b}}^{\text{ P}}(\tau )$ , if and only if

(8) $$\begin{array}{r} (1-p(\tau ))(F+c+h-{\underline{b}}^{\text{ P}}(\tau ))-p(\tau )({\underline{b}}^{\text{ P}}(\tau )+B\left(1-\delta \right)+w)\geq 0. \end{array}$$

Substituting ${\underline{b}}^{\text{ P}}(\tau )$ from equation (6), we observe that the payer would be willing to provide bonus ${\underline{b}}^{\text{ P}}(\tau )$ if and only if the provider’s opportunity cost $k(\tau )<{\hat{k}}^{\text{ P}}(\tau )$ . Note that the condition is independent of the visit fee w and is the same condition under which the central planner will not prescribe antibiotics. The payer’s expected gain of avoiding over-prescribing is decreasing in τ because the probability of bacterial infection is increasing in the symptom level. Therefore, we can find the symptom threshold τ* , as presented in Proposition 2.

Proposition 2 When diagnosis relies on clinical interpretation of symptom presentation, the payer sets the visit fee w to absorb all the surplus (i.e., $\Pi_{P}={\underline{\Pi }}_{P}$ ) and

1. if $k(0)>{\hat{k}}^{\text{ P}}(0)$ , the payer offers a bonus smaller than ${\underline{b}}^{\text{ P}}(0)$ ; the provider prescribes antibiotics to all patients (i.e., τ*=0 ),
2. if $k(0)\leq {\hat{k}}^{\text{ P}}(0)$ the payer offers a bonus equal to ${\underline{b}}^{\text{ P}}(\tau *)$ ; the provider prescribes antibiotics if and only if a patient’s symptom level is above the threshold $\tau *=\arg\;\left\{0\leq \tau \leq 1:p(\tau )=\frac{\left(1+\alpha )F+c+h-k(\tau \right)}{\left(1+\alpha )F+c+h+(1+\alpha )B(1-\delta \right)}\right\}$ .

When the opportunity cost the provider incurs by not prescribing antibiotics to a patient with the lowest bacterial-type symptoms, k(0) , is larger than ${\hat{k}}^{\text{ P}}(0)$ , the benefit of reducing inappropriate antibiotic prescriptions for viral infections does not justify financial incentives. Therefore, the payer offers a bonus less than the minimum required bonus and the provider prescribes antibiotics to all patients. In contrast, when $k(0)\leq {\hat{k}}^{\text{ P}}(0)$ , the payer offers the minimum bonus required to incentivize the provider to apply the same antibiotic prescription behavior as in the integrated care system. Thus, we show that when the opportunity cost of the provider is public information, the payer can coordinate the care using an action-based incentive payment model, achieving the socially optimal antibiotic prescription policy (i.e., first-best policy).

Corollary 1 confirms that a payment contract with only a per-visit fee ( w ) cannot achieve the first-best policy resulting in sub-optimal diagnosis and antibiotic prescription behaviour, as observed in practice.

Corollary 1 When diagnosis relies on clinical interpretation of symptom presentation,

• when $k(0)>{\hat{k}}^{\text{ P}}(0)$ , an action-based payment contract (i.e., ζ={w,b} ) results in the same outcome as single visit fee contract (i.e., ζ={w} ),
• when $k(0)\leq {\hat{k}}^{\text{ P}}(0)$ , an action-based payment contract results in either

∘

fewer inappropriate antibiotic prescriptions,

∘

or a lower cost to the payer.

With an action-based payment contract (i.e., ζ={w,b} ), when the opportunity cost of the patient with the lowest opportunity cost is larger than ${\hat{k}}^{\text{ P}}(0)$ (i.e., $k(0)>{\hat{k}}^{\text{ P}}(0)$ ), the payer does not provide the minimum required bonus payment to avoid unnecessary antibiotic prescriptions and thus all patients will be prescribed with antibiotics. Therefore, the action-based payment contract does not offer an advantage over a single visit fee benchmark, which represents the current practice. However, when it is socially optimal not to prescribe antibiotics to some of the patients diagnosed with a viral infection (i.e., $k(0)\leq {\hat{k}}^{\text{ P}}(0)$ ), an action-based payment contract always results in a better outcome compared to a single visit fee contract. This is because, with a single payment contract, the payer can either absorb the surplus of the provider (i.e., $\Pi_{p}={\underline{\Pi }}_{P}$ ) with the visit fee and allow the provider to prescribe antibiotics to patients with a viral diagnosis or pay some surplus to avoid unnecessary antibiotic prescriptions.

Note that, in our base model, we do not include the social harm of antibiotic resistance in the provider’s utility function. In a sensitivity analysis, we included the social harm of antibiotics in the provider’s reward function to evaluate the robustness of our findings (details of such analysis are presented in the E-companion). If provider’s do weigh the social harm of antibiotic resistance in their decision making, fewer patients are prescribed antibiotics and a smaller bonus is necessary to further reduce antibiotic prescribing. Despite these changes, our structural findings remain robust.

## 5. Optimal Contract When Diagnosis Relies on Additional Testing

In this section, we consider the scenario when the diagnosis is informed by the use of a technical diagnostic test, for example, rapid antigen detection or throat culture for the evaluation of Group A streptococcus infection in patients presenting with a sore throat. We assume the diagnostic accuracy of the technical diagnostic is exogenously fixed and characterized by sensitivity, denoted $q_{1}$ , and specificity, denoted $q_{2}$ , where sensitivity is the probability of correctly diagnosing a bacterial infection and specificity is the probability of correctly diagnosing a viral infection. Further, we assume $q_{1}+q_{2}>1$ , indicating a properly labeled test and to ensure that a random diagnosis based on the prevalence of each infection type would not achieve a higher diagnostic accuracy. Under such assumptions, the probabilities of the four possible health outcomes defined in Section 3.2 can be written as follows:

(9) $$\begin{array}{rl} P_{A}(s,\tau ,q_{1},q_{2}) & =p(s)q_{1}+I_{\tau \leq s}\;p(s)(1-q_{1}),\\ P_{B}(s,\tau ,q_{1},q_{2}) & =\left(1-p(s)\right)\left(1-q_{2}\right)+I_{\tau \leq s}\;\left(1-p(s)\right)q_{2},\\ P_{C}(s,\tau ,q_{1},q_{2}) & =I_{s\leq \tau }\;p(s)\left(1-q_{1}\right),\\ P_{D}(s,\tau ,q_{1},q_{2}) & =I_{s\leq \tau }\;\left(1-p(s)\right)q_{2}. \end{array}$$

Optimizing over the provider’s prescription threshold, τ , when diagnosis relies on additional testing, the payer’s problem is as follows:

(OPT2) $$\begin{array}{rl} & \max_{\zeta \in R^{2}}\;\Pi_{S}(P\left(\tau *\right),B,F,c,h,\zeta )\\ \text{s.t.} & \{\tau *\}=\underset{\tau \in \left[0,1\right]}{\arg\;\max}\;\Pi_{P}(P\left(\tau \right),B,F,k,\zeta )\\ & \Pi_{P}(P\left(\tau *\right),B,F,k,\zeta )\geq {\underline{\Pi }}_{P}. \end{array}$$

Following the same structure as in the last section, we first analyse optimal antibiotic prescription behavior in an integrated care system as a benchmark (Section 5.1) and then we evaluate a decentralized care system where the provider’s antibiotic prescription behavior depends on the contract payment terms offered by the payer (Section 5.2).

### 5.1. Integrated Care System

The central planner may decide not to prescribe antibiotics to a viral diagnosis to avoid over-prescribing (in the case of TV), at the cost of increasing the risk of under-prescribing (in the case of FV). When using a technical diagnostic, the chance of a TV is the posterior probability of a viral infection, conditioned on the test indicating a viral infection, that is, $\frac{(1-p(\tau ))q_{2}}{\left(1-p(\tau ))q_{2}+p(\tau )(1-q_{1}\right)}$ . Similarly, we calculate the likelihood of a FV diagnosis. Utilizing the cost estimates detailed in Section 4.1, the central planner aims to strike a balance between the expected cost of over- and under-prescribing. Therefore, the central planner will not prescribe antibiotics to a patient diagnosed with a viral infection at symptom level τ if $k(\tau )\leq {\hat{k}}^{\text{ T}}(\tau )$ , where

(10) $$\begin{array}{rl} {\hat{k}}^{\text{ T}}(\tau ) & =\frac{(1-p(\tau ))q_{2}}{\left(1-p(\tau ))q_{2}+p(\tau )(1-q_{1}\right)}((1+\alpha )F+c+h)\\ & \;-\frac{p(\tau )(1-q_{1})}{\left(1-p(\tau ))q_{2}+p(\tau )(1-q_{1}\right)}(1+\alpha )B(1-\delta ) \end{array}$$

is the provider’s opportunity cost threshold for patients at symptom level τ . Since k(τ) is increasing and ${\hat{k}}^{\text{ T}}(\tau )$ is decreasing in symptom level τ , we can find the optimal prescription threshold as presented in Proposition 3.

Proposition 3 When diagnosis relies on technical diagnostic test, in an integrated care system,

1. if $k(0)>{\hat{k}}^{\text{ T}}(0)$ , the provider prescribes antibiotics to all patients diagnosed with a viral infection (i.e., τ*=0 ),
2. if $k(0)\leq {\hat{k}}^{\text{ T}}(0)$ , the provider prescribes antibiotics to a patient with viral diagnosis if and only if the patient’s symptom level is above the symptom threshold τ* where
τ*=arg{q2((1+α)F+c+h−k(τ))q2((1+α)F+c+h−k(τ))+(1−q1)((1+α)B(1−δ)+k(τ))0≤τ≤1:p(τ)=q2((1+α)F+c+h−k(τ))q2((1+α)F+c+h−k(τ))+(1−q1)((1+α)B(1−δ)+k(τ))}.

Threshold ${\hat{k}}^{\text{ T}}(\tau )$ is influenced by diagnostic uncertainty, specifically the post-test probability of bacterial infection among patients diagnosed with a viral infection. In particular, at any symptom level τ , ${\hat{k}}^{\text{ T}}(\tau )$ increases in the probability of a TV diagnosis and decreases in the probability of FV diagnosis. This shows that a test with greater diagnostic accuracy increases ${\hat{k}}^{\text{ T}}(\tau )$ thus it decreases the frequency of inappropriate antibiotic prescription. This is in contrast to the case where diagnosis relies on clinical interpretation of symptom presentation (Section 4) where the diagnostic decision could be affected by the provider’s interest.

### 5.2. Decentralized Care System

In this section, we present the optimal solution for a decentralized care system where the provider’s optimal antibiotic prescription behavior is in response to the contract payment terms offered by the payer, as well as the diagnostic accuracy. Following the same logic as in Section 4.2, the provider’s response to a payment contract ζ={w,b} is to not prescribe antibiotics to a patient diagnosed with viral infection when the expected gain of avoiding over-prescribing is higher than the expected loss of under-prescribing. Specifically, the provider will not prescribe antibiotics to a patient at symptom level τ who is diagnosed with a viral infection if $b\geq {\underline{b}}^{\text{ T}}(\tau )$ , where

(11) $$\begin{array}{rl} {\underline{b}}^{\text{ T}}(\tau ) & =-\frac{\left(1-p(\tau )\right)q_{2}}{\left(1-p(\tau )\right)q_{2}+p(\tau )\left(1-q_{1}\right)}(\alpha F-k(\tau ))\\ & \;+\;\frac{p(\tau )\left(1-q_{1}\right)}{\left(1\;-\;p(\tau )\right)q_{2}\;+\;p(\tau )\left(1\;-\;q_{1}\right)}\\ & \;\times (\alpha B(1\;-\;\delta )\;+\;k(\tau )\;-\;w). \end{array}$$

Lemma 3 When diagnosis relies on a technical diagnostic test, given a payment contract ζ={w,b} , the provider prescribes antibiotics to a patient with a diagnosis of viral infection if and only if the patient’s symptom level is above the threshold $\tau^{*}\left(w,b\right)$ where

(12) τ*(w,b)={0if b<b_ T(0);arg{=q2(αF−k(τ)+b)(1−q1)(αB(1−δ)+k(τ)−w−b)+q2(αF−k(τ)+b)}0≤τ≤1:p(τ)=q2(αF−k(τ)+b)(1−q1)(αB(1−δ)+k(τ)−w−b)+q2(αF−k(τ)+b)}if b_ T(0)≤b≤b_ T(1);1if b>b_ T(1).

Lemma 3 defines ${\underline{b}}^{\text{ T}}(\tau )$ as the minimum reward payment, at a given visit fee w , that the provider requires to not prescribe antibiotics to a patient at symptom level τ who is diagnosed with a viral infection. This minimum reward payment compensates the provider’s expected benefit from prescribing antibiotics to those patients (i.e., avoiding under-prescription, as well as the opportunity cost).

Knowing the provider’s response, the payer balances their cost of over- and under-prescribing by providing the minimum bonus ${\underline{b}}^{\text{ T}}(\tau )$ to prevent antibiotic prescription to patients at symptom level τ who are diagnosed with viral infection if and only if

(13) $$\begin{array}{rl} & \frac{\left(1-p(\tau )\right)q_{2}}{\left(1-p(\tau )\right)q_{2}+p(\tau )\left(1-q_{1}\right)}(F+c+h-{\underline{b}}^{\text{ T}}(\tau ))\\ & \;-\frac{p(\tau )\left(1-q_{1}\right)}{\left(1-p(\tau )\right)q_{2}+p(\tau )\left(1-q_{1}\right)}({\underline{b}}^{\text{ T}}(\tau )+B(1-\delta )+w)\geq 0. \end{array}$$

Substituting ${\underline{b}}^{\text{ T}}(\tau )$ from equation (11) into equation (13), we observe that, independent of visit fee w , the payer provides incentives when $k(0)\leq {\hat{k}}^{\text{ T}}(0)$ . This is the same condition under which antibiotics are not prescribed in an integrated care system (Proposition 3). Predicting the behavior of the provider, the payer identifies the set of optimal contract payments ζ={w,b} as presented in Proposition 4.

Proposition 4 When diagnosis relies on a technical diagnostic test, the payer sets the visit fee w to absorb all the surplus (i.e., $\Pi_{P}={\underline{\Pi }}_{P}$ ) and

1. if $k(0)>{\hat{k}}^{\text{ T}}(0)$ , the payer offers a bonus smaller than ${\underline{b}}^{\text{ T}}(0)$ ; the provider prescribes antibiotics to all patients diagnosed with a viral infection (i.e., τ*=0 ),
2. if $k(0)\leq {\hat{k}}^{\text{ T}}(0)$ , the payer offers a bonus equal to ${\underline{b}}^{\text{ T}}(\tau *)$ ; the provider prescribes antibiotics to a patient diagnosed with a viral infection if and only if the patient’s symptom level is above the threshold τ*=arg{q2((1+α)F+c+h−k(τ))(q2((1+α)F+c+h−k(τ))+(1−q1)((1+α)B(1−δ)+k(τ))}0≤τ≤1:p(τ)=q2((1+α)F+c+h−k(τ))(q2((1+α)F+c+h−k(τ))+(1−q1)((1+α)B(1−δ)+k(τ))} .

Similar to the case when diagnosis relies on clinical interpretation of symptom presentation, when the provider’s opportunity cost for the patient at lowest symptom level, k(0) is higher than the threshold ${\hat{k}}^{\text{ T}}(0)$ , the payer sets the bonus payment to a level lower than the minimum required by the provider, including b=0 . In this case (Proposition 4.1), the provider will continue to prescribe antibiotics to patients diagnosed with viral infection. For providers with lower opportunity costs (Proposition 4.2), the payer sets the bonus payment to compensate precisely for the provider’s expected benefit from prescribing antibiotics for viral diagnosis. As a result, a provider with $k(0)\leq {\hat{k}}^{\text{ T}}(0)$ will not prescribe antibiotics for patients diagnosed with viral infection if their symptom level is lower than the threshold τ* . In either case, the payer absorbs all the surplus of the provider (i.e., $\Pi_{p}={\underline{\Pi }}_{P}$ ) with the visit fee. Therefore, when diagnosis relies on technical diagnostic test, the payer can coordinate the care using an action-based incentive payment model and achieve the first-best policy with the same antibiotic prescription behavior and corresponding payoff as in an integrated care system.

To understand the impact of using a technical diagnostic test on antibiotic prescription decisions, Corollary 2 compares the proportion of true viral patients receiving antibiotics with or without bacterial testing.

Corollary 2 All else equal, the use of a technical diagnostic test reduces the rate of inappropriate antibiotic prescription when the test has high specificity (i.e., $q_{2}\geq \underline{q_{2}}$ ).

The action-based incentive payment model ensures socially optimal antibiotic prescription levels, both with or without the use of a technical diagnostic test (Propositions 2 and 4). However, Corollary 2 shows that an accurate test can further reduce inappropriate antibiotic prescription by addressing misdiagnosis concerns and the associated costs of misdiagnosis for the payer and provider. We show that compared to the case where diagnosis relies on clinical interpretation of symptoms, the antibiotic prescription threshold is higher when diagnosis relies on a technical diagnostic test, because in this case the provider is unable to alter the diagnosis. Therefore, to reduce inappropriate antibiotic prescription it is sufficient for the test to accurately identify patients who have viral infection (i.e., $q_{2}\geq \underline{q_{2}}$ ).

Corollary 3 confirms that a payment contract with only a per-visit fee ( w ) cannot achieve the first-best policy even when diagnosis relies on a technical diagnostic.

Corollary 3 When diagnosis relies on a technical diagnostic test,

• if $k(0)>{\hat{k}}^{\text{ T}}(0)$ , an action-based payment contract (i.e., ζ={w,b} ) results in the same outcome as single visit fee contract (i.e., ζ={w} ),
• if $k(0)\leq {\hat{k}}^{\text{ T}}(0)$ , an action-based payment contract results in either

∘

fewer inappropriate antibiotic prescriptions, or

∘

lower cost to the payer.

## 6. Case Study

We apply our framework to patients presenting to primary care providers with a sore throat. Antibiotics are indicated in approximately 10%–15% of cases, when patients have a GABHS infection (“strep throat”), but antibiotics are prescribed in ∼ 70% of cases (Worrall et al., 2007; Linder and Stafford, 2001). Providers can rely on the presentation of clinical symptoms to classify patients as having a GABHS infection (requiring antibiotics), or another infection, primarily viral, for which antibiotics are not indicated. Clinical guidelines recommend the use of rapid antigen tests or throat culture to aid in the diagnosis of patients with moderate probability (10%–40%) of GABHS infection rather than relying on clinical presentation alone (Kalra et al., 2016). In this section, we apply our framework first to the case where diagnosis relies on clinical interpretation of symptom presentation to demonstrate the influence a bonus payment can have on the provider’s diagnostic criteria. Then, we demonstrate how a bonus can achieve the first-best policy with the use of a technical diagnostic test.

A detailed exposition of all assumptions and inputs for the case study are presented in E-companion. As stated earlier, we assume the overall prevalence of bacterial infection is 10%, that is, E(p)=10% , consistent with the overall rate of GABHS infection in patients presenting with sore throat. For the purpose of illustrating additional properties of the solution, we also present results for E(p)=40% .

### 6.1. Diagnosis Relies on Symptom Presentation

Without the rapid test, diagnosis of GABHS relies on an assessment of clinical symptoms. Consistent with high observed rates of antibiotic prescriptions to patients presenting with sore throat in practice (Linder and Stafford, 2001; Humair et al., 2006; McIsaac et al., 2004), the opportunity cost associated with not prescribing antibiotics incentivizes physicians to prescribe antibiotics to a larger group of patients than are clinically indicated. Based on the rewards and probabilities of each outcome, the patient, the payer, and society strictly prefers a policy of no antibiotics to patients diagnosed with viral infections, but the provider prefers a policy of prescribing antibiotics to patients diagnosed with a viral infection.

**Figure 5.**
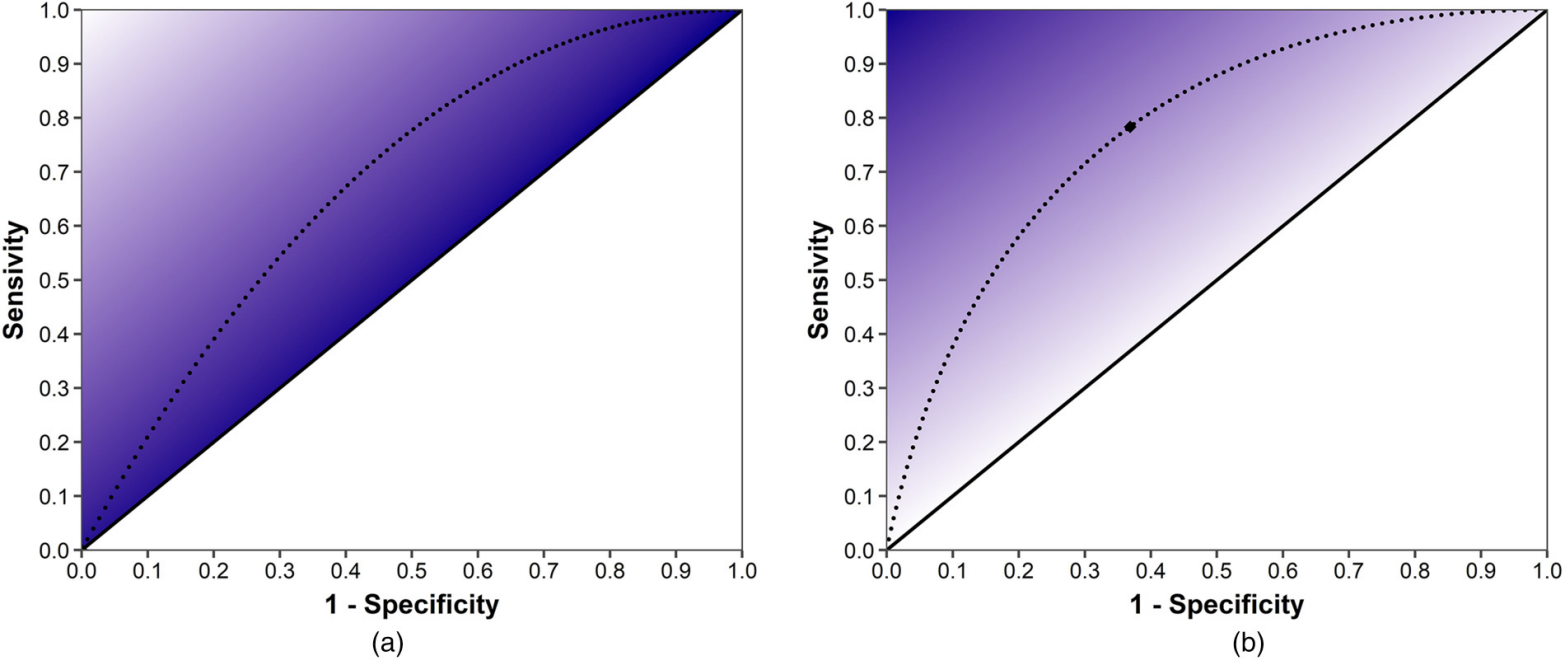
Provider’s relative reward at various diagnostic thresholds in response to bonus contract. Solid line represents where sensitivity equals specificity; diagnostic accuracy below this line would indicate a mislabeled diagnostic. Dotted line represents the receiver–operator curve. Providers can move their diagnostic accuracy along this curve; τ=0 occurs at $\left(q_{1},1-q_{2})=(1,1\right)$ and τ=1 occurs at $\left(q_{1},1-q_{2})=(0,0\right)$ . Darker shading represents higher expected reward from the provider’s perspective. The filled diamond represents the provider’s optimal diagnostic threshold when offered the optimal contract $\zeta^{*}$ . (a) E(p)=10% ; and (b) E(p)=40% .

We illustrate the provider’s expected reward for various τ when E(p)=10% in Figure 5(a). In this case, the payer offers $\zeta^{*}=(w=\$31.51,b=\$35.15)$ . This contract offer is the minimum acceptable bonus to the provider such that the provider is indifferent between providing antibiotics to all patients, that is, $\left(q_{1},q_{2})=(1,0\right)$ , and providing antibiotics to no patients, that is, $\left(q_{1},q_{2})=(0,1\right)$ . Therefore, from any initial diagnostic threshold, the provider shifts their diagnostic accuracy to diagnose no patients with bacterial infection. Because diagnosing no patients with bacterial infections may not be professionally acceptable, the provider will move their diagnostic criteria to the professional boundary closest to this action.

We present the case of E(p)=40% in Figure 5(b) to illustrate additional properties of the solution. In this case, the payer offers $\zeta^{*}=(w=\$9.88,b=\$37.63)$ . This contract offer leads to an optimal prescription threshold $\tau^{*}$ corresponding to $\left(q_{1},q_{2})=(78.3\%,63.1\%\right)$ such that the provider does not prescribe antibiotics to patients with symptom level less than $\tau^{*}$ . Therefore, from any initial diagnostic threshold, the provider shifts their diagnostic accuracy to this point (over)aligning their prescribing behavior to the incentive created by the bonus. In this case, the visit fee is much lower than the current visit fee ($37.50) and the optimal visit fee when E(p)=10% to ensure the provider is not enticed by the second visit fee in patients with bacterial infection who are initially mis-diagnosed and denied antibiotics.

### 6.2. Diagnosis Relies on Additional Testing

A rapid antigen test is recommended for patients presenting with a sore throat who have an intermediate modified Centor scores of 1, 2, or 3 (Choby, 2009). With the rapid antigen test, more patients receive an accurate diagnosis. However, providers are still inclined to prescribe antibiotics to patients diagnosed with a viral infection to avoid the opportunity cost incurred when taking the time to explain that antibiotics are not indicated for viral infections and patient disappointment when their provider does not provide expected treatment. This inclination to prescribe antibiotics to patients diagnosed with viral infections remains true as long as the provider’s opportunity cost exceeds $0.80 . Using Proposition 4, we calculate the optimal contract terms when E(p)=10% to be $\zeta^{*}=(w=\$31.39,b=\$37.15)$ . With these terms, the provider stops prescribing antibiotics to patients diagnosed with a viral infection based on the results of the rapid antigen test.

**Figure 6.**
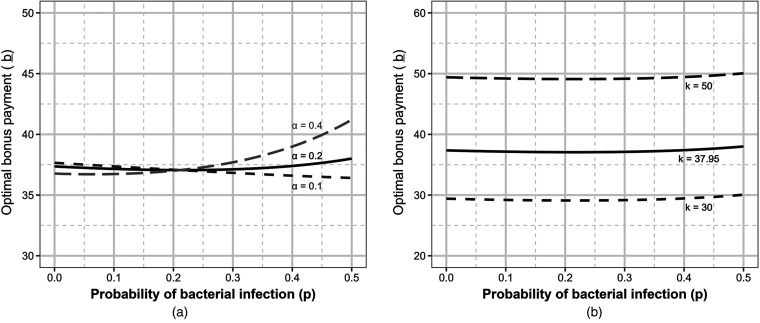
Sensitivity analysis on the optimal bonus payment when diagnostic accuracy relies on a technical diagnostic test, varying the probability of bacterial infection ( p ), provider altruism ( α ), and provider opportunity cost ( k ). (a) Optimal bonus payment, varying p and α ; and (b) optimal bonus payment, varying p and k .

Sensitivity analysis demonstrates that the optimal bonus payment is robust to the prevalence of bacterial infection and to the provider’s level of altruism (Figure 6(a)). However, the optimal bonus payment is highly sensitive to the provider’s opportunity cost (Figure 6(b)). Using equation (10), we find that there is a coordinating contract acceptable to both the payer and the provider as long as the provider’s opportunity cost is < $94.55.

### 6.3. Policy Insights

Rapid antigen tests are a low-cost diagnostic already recommended for use in patients presenting with sore throat who, based on their symptom profile, have a moderate probability (10%–40%) of GABHS infection (Kalra et al., 2016). Rapid tests have greater sensitivity and specificity compared to clinical assessment, remove substantial subjective judgement from the clinical diagnosis process, and do not require waiting 24-hours for the results of a throat culture. Bonus payments incentivize physicians to not prescribe antibiotics to patients they diagnose with a viral infection. However, when diagnosis relies on the clinical interpretation of symptom presentation, bonus payments also incentivize providers to shift their diagnostic accuracy, increasing the number of patients diagnosed with a viral infection, potentially to the clinical detriment of patients who truly have bacterial infections. A high-quality point-of-care diagnostic takes time to use which may extend the duration of a patient’s visit, but the results of the diagnostic may support the provider’s efforts to explain why they are not prescribing antibiotics. Ultimately, use of a high-quality diagnostic tool protects the health of patients with bacterial infections when implementing an incentive payment to reduce inappropriate antibiotic prescribing.

**Figure 7.**
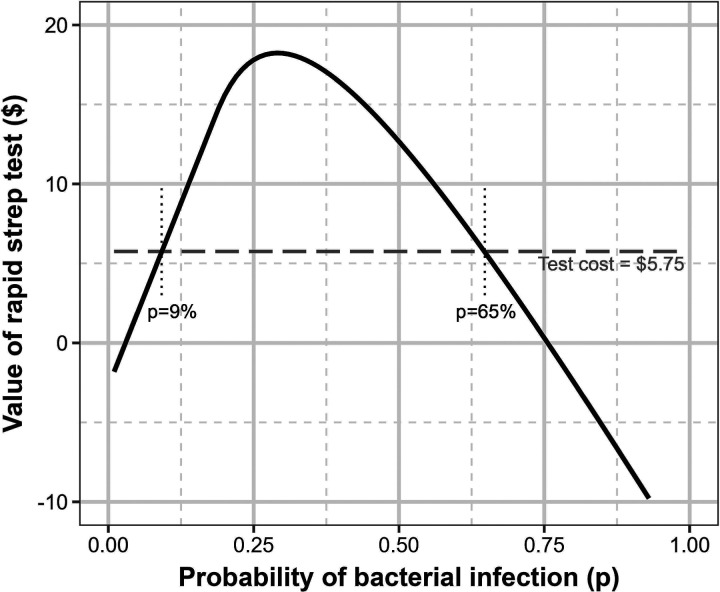
Value of a rapid strep test, varying the probability of bacterial infection ( p ).

Specific to this example, a rapid strep test costs the payer $5.70 (Ontario Ministry of Health and Long Term Care, 2023) and an antibiotic prescription costs $12.55. We calculated the value of the test to the payer by comparing the expected value of outcomes, using the optimal bonus, with and without the rapid strep test. We identify that the test value exceeds the current cost of providing the test ($5.75), for patients with expected pre-test probability of bacterial infection between 9% and 65% (Figure 7), which is a broader range than is currently recommended. Therefore, it is cost-effective to insist providers use this low-cost point of care test for the majority of patients presenting with sore throat with a contract optimized for the diagnostic accuracy of the test, rather than offer a contract optimized for the poorer diagnostic accuracy of physician assessment alone.

## 7. Extension: Opportunity Cost as Private Information

So far, we assumed that the opportunity cost is public information. However, as discussed in Section 3.3, there might be heterogeneity in the expected opportunity cost of providers because of differences in factors such as physician training, fear of misdiagnosis, challenges communicating with patients, and time pressure. This results in different perceived opportunity cost by providers that cannot be known or observed by the payer. Therefore, in this section, we study the impact of private information of the providers about their opportunity cost on the application of incentive payments in achieving the socially optimal levels of antibiotic prescription defined in Sections 4.1 and 5.1.

We assume that the provider can be one of two types: low opportunity cost (type- L ), with probability $\beta_{L}$ , or high opportunity cost (type- H ), with probability $\beta_{H}=1-\beta_{L}$ . The provider’s opportunity cost is denoted by $k_{i}(\tau )$ , i∈{L,H} , where $k_{H}(\tau )>k_{L}(\tau )\geq 0$ for any τ∈[0,1] . The payer knows the distribution of provider types, but the opportunity cost of a specific provider is private information to that provider, unobserved by the payer.

First, in Section 7.1, we extend our analysis to situations where the payer offers a single contract to both provider types. Then, in Section 7.2, we consider offering menu of contracts to providers.

### 7.1. Single Contract for Both Provider Types

To incorporate the two types of providers, we update the payer’s problem when diagnosis relies on clinical interpretation of symptoms, defined in (OPT1), as shown in (OPT3).

(OPT3) $$\begin{array}{rl} & \max_{\zeta \in R^{2}}\;\sum_{i\in \{L,H\}}\beta_{i}\;\Pi_{S}(P_{i}\left(\tau_{i}*,\theta_{i}*\right),B,F,c,h,\zeta )\\ \text{s.t.} & \{\tau_{i}*,\theta_{i}*\}=\underset{0\leq \tau_{i}\leq \theta_{i}\leq 1}{\arg\;\max}\;\Pi_{P_{i}}(P_{i}\left(\tau_{i},\theta_{i}\right),B,F,k_{i},\zeta )\\ & \Pi_{P_{i}}(P_{i}\left(\tau_{i}*,\theta_{i}*\right),B,F,k_{i},\zeta )\geq {\underline{\Pi }}_{P_{i}}\;i\in \{L,H\}. \end{array}$$

The model where diagnosis relies on additional use of a technical diagnostic test, (OPT2) can be updated accordingly. In either case, each provider type sets the prescription threshold independently based on their private opportunity cost. Therefore, the minimum reward payment for type- i∈{L,H} provider relies on symptom presentation or additional testing follows from equation (6) or equation (11), respectively, by substituting k(τ) with $k_{i}(\tau )$ . Subsequently, the response of each provider type to the payment contract ζ={w,b} follows from Lemma 2 (symptom presentation) or Lemma 3 (additional testing) with the corresponding bonus threshold for each provider type.

**Figure 8.**
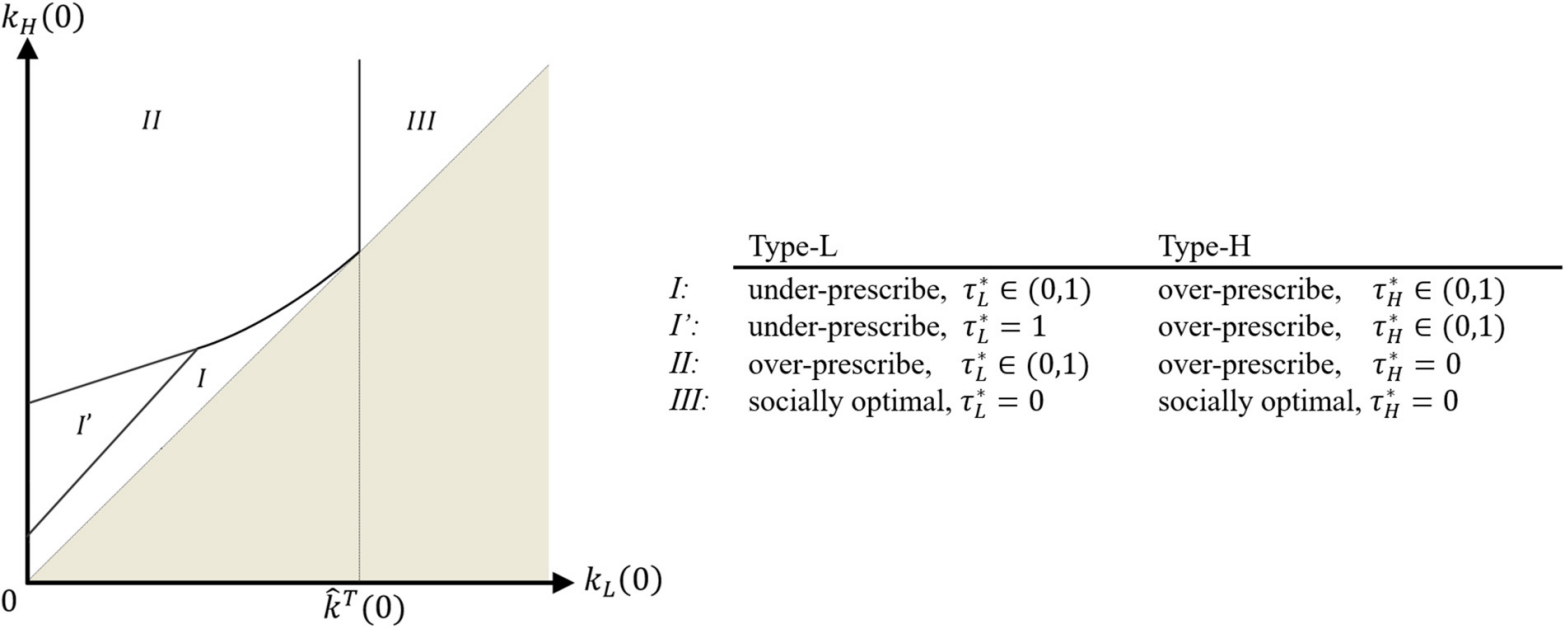
Antibiotic prescription behavior of provider when opportunity cost is private information.

We illustrate the behavior induced by the optimal contract for the case where the diagnosis relies on additional diagnostic testing in Figure 8 (using the parameters of the case study in Section 6, and assuming both opportunity costs $k_{H}(\tau )$ and $k_{L}(\tau )$ are linearly increasing in symptom level with the same slope). Below we discuss the intuition behind how the optimal prescription behavior changes with respect to $k_{H}(0)$ and $k_{L}(0)$ . The insights are the same for case that diagnosis relies on symptom presentation, as well as other parameterizations for the model, including normal and uniform distributions for $f_{s}(.)$ function.

When $k_{L}(0)=k_{H}(0)\leq {\hat{k}}^{T}(0)$ the system is equivalent to the one with public information. Therefore, it follows from Proposition 4 that the payer offers bonus ${\underline{b}}^{T}(\tau *)={\underline{b_{H}}}^{T}(\tau {*}_{H})={\underline{b_{L}}}^{T}(\tau {*}_{L})$ so both providers will follow the socially optimal prescription behavior. However, when $k_{L}(0)<k_{H}(0)$ , the payer needs to balance the conflicting incentives of the two provider types: the type- H provider is leaning toward over-prescribing to avoid incurring the opportunity cost, and the type- L provider may benefit from information rent by under-prescribing comparing to the socially optimal prescription level. To incentivize both provider types to prescribe antibiotics only to some patients (Figure 8 Region I, $0<\tau_{H}*\leq \tau_{L}*<1$ ), the payer offers a bonus $b\geq {\underline{b_{H}}}^{T}(0)$ . This contract becomes less attractive for the type- H and more attractive for type- L provider at higher $k_{H}$ values, that is, we observe lower $\tau_{H}*$ and higher $\tau_{L}*$ . Ultimately, this trade-off may reach one of the following two limits: (i) incentivizing the type- H provider becomes too costly, thus, the payer decides to focus on the type- L provider by offering a bonus ${\underline{b_{L}}}^{T}(0)\leq b<{\underline{b_{H}}}^{T}(0)$ (move from Region I to Region II, $0<\tau_{L}*<1$ ; $\tau_{H}*=0$ ), or (ii) the bonus becomes high enough for the type- L provider to not prescribe antibiotics to *any* patient with viral test results (move from Region I to Region I′, $0<\tau_{H}*<1$ and $\tau_{L}*=1$ ). In this case, at higher $k_{H}(0)$ values, eventually we reach a point that the payer decides to focus only on the type- L provider (move from Region I′ to II). Finally, if $k_{L}(0)>{\hat{k}}^{T}(0)$ , the payer offers a bonus $b<{\underline{b_{L}}}^{T}(0)$ , therefore, both provider types choose to prescribe antibiotics to *all* patients with viral test results (Region III, $\tau_{H}*=\tau_{L}*=0$ ).

#### 7.1.1. Efficiency of a Single Contract

A single contract needs to balance the incentive of type- H provider with the information rent of type- L provider. Particularly, in Region I of Figure 8, incentivizing type- H provider to reduce over-prescribing, pushes the prescription threshold of type- L provider towards under-prescribing in order to earn more information rent. The same inefficiency can be observed in Region I′ of Figure 8, where the under-prescribing behavior of type- L provider has reached its limit, that is, $\tau_{L}*=1$ . Therefore, the payer can offer a higher bonus to decrease the level of over-prescribing by type- H provider, without the fear of worsening type- L provider’s antibiotic prescription behavior. This means in Region I′, the type- L provider’s antibiotic prescription behaviour is further from the first-best policy, but type- H provider behavior is closer, compared to Region I. In either case, the payer cannot coordinate care with an action-based contract. A single contract also shows inefficiency in Region II of Figure 8, where type- H provider prescribes antibiotics to all patients, even at opportunity costs lower than ${\hat{k}}^{T}(0)$ . Moreover, to satisfy the individual rationality of type- H provider, the payer needs to provide a higher visit fee and a lower bonus. Therefore, type- L provider also over-prescribes compared to the socially optimal antibiotic prescription levels. Finally, Region III illustrates the improbable case that the opportunity cost of both provider types is extremely high. Under such circumstances, the first-best policy is to prescribe antibiotics to everyone, which can be achieved by not providing a bonus.

Remark 5 Throughout our analysis in this section, we assumed that there are only two types of providers which is a common assumption in the literature (e.g., Yaesoubi and Roberts, 2011; Cakanyıldırım et al., 2012; Xiao and Xu, 2012). However, extending the analysis to multiple types follows directly. The unique opportunity costs of providers can be ordered such that $k_{1}<\cdots <k_{n-1}<k_{n}$ , where $k_{1}$ is the provider with the lowest opportunity cost and $k_{n}$ is the provider with the highest opportunity cost. It is straightforward to show that the payer can identify $\hat{k}(\tau )$ based on the distribution of provider opportunity costs. Provided with the payment contract, providers with higher opportunity cost will over-prescribe in response to the contract, and providers with lower opportunity cost will under-prescribe and will have positive surplus (information rent).

### 7.2. Menu of Contracts

The payer may consider offering a menu of contracts to achieve coordinated care while learning the provider’s true type (i.e., opportunity cost). In this section, we extend our analysis to evaluate how menu of contracts impact outcomes when bonus payments can affect the diagnosis behavior of the provider. In particular, the payer’s problem becomes determining a menu of contracts $\zeta_{i}=\{w_{i},b_{i}\}$ to solve the following optimization problem when diagnosis relies on interpretation of symptom presentation.

(OPT4) $$\begin{array}{rl} & \max_{\zeta_{i}\in R^{2}}\;\sum_{i\in \{L,H\}}\beta_{i}\;\Pi_{S}(P_{i}\left(\tau_{i}*,\theta_{i}*\right),B,F,c,h,\zeta_{i})\\ \text{s.t.} & \{\tau_{i}*,\theta_{i}*\}=\underset{\tau_{i},\theta_{i}\in \left[0,1\right]}{\arg\;\max}\;\Pi_{P_{i}}(P_{i}\left(\tau_{i},\theta_{i}\right),B,F,k_{i},\zeta_{i})\\ & \Pi_{P_{i}}(P_{i}\left(\tau_{i}*,\theta_{i}*\right),B,F,k_{i},\zeta_{i})\\ & \;\geq \max_{\tau_{i},\theta_{i}\in \left[0,1\right]}\Pi_{P_{i}}(P_{i}\left(\tau_{i},\theta_{i}\right),B,F,k_{i},\zeta_{j})\\ & \Pi_{P_{i}}(P_{i}\left(\tau_{i}*,\theta_{i}*\right),B,F,k_{i},\zeta_{i})\geq {\underline{\Pi }}_{P_{i}}\;i,j\in \{L,H\}. \end{array}$$

The model where diagnosis relies on a technical diagnostic test, (OPT2) can be updated accordingly. The first set of constraints ensure that the provider’s optimal treatment choice is $\left\{\tau_{i}*,\theta_{i}*\right\}$ . The second set of constraints are the provider’s adverse selection incentive-compatibility (IC) constraints. These constraints ensure that the type- i provider prefers contract $\zeta_{i}$ over the contract for the other provider type and, thus, prevents provider misrepresentation. Note that the right-hand-side of the IC constraints take into account that a provider may choose to misrepresent themselves which may influence their treatment decisions. The third set of constraints ensure the provider’s participation.

This problem is not mathematically tractable because the provider can change the proportion of the patients receiving antibiotics by changing the prescription threshold that is their private information (moral hazard). Therefore, when applying the revelation principle, a menu of contracts may not guarantee IC. Specifically, when switching between the contracts, the provider can always maintain the outcome from the other contract by adjusting their prescription threshold. Through extensive numerical studies, we observe that when the optimal diagnostic threshold is an interior solution (i.e., $0<\tau_{i}^{*}<1$ , Region I in Figure 8) a menu of contracts cannot outperform the single contract. However, in cases where at least one provider type chooses an extreme prescription threshold, offering a menu of contracts can improve the outcome. In particular, a menu of contracts can achieve socially optimal prescription levels in Region II and decrease the over-prescribing of type- H provider in Region I′. As a result, offering a menu of contracts makes Region I smaller. Except for this one case, all the substantive insights drawn from the single contract remain unchanged. Therefore, we conclude that, applying menu of contracts that is more complicated to implement would not significantly improve the outcome of the system.

## 8. Conclusion and Managerial Implications

Overuse and misuse of antibiotics significantly contributes to antibiotic resistance and adverse patient outcomes. In this study, we examine the implications of incentive payments on primary care physician antibiotic prescription behavior. In particular, we develop a physician compensation model to study the interaction between a healthcare payer and a care provider who makes the antibiotic prescription decision for patients presenting with symptoms potentially representing a viral or bacterial infection. To maximize social welfare, the payer offers an action-based payment contract to providers, which includes a bonus payment for not prescribing antibiotics to patients diagnosed with a viral infection. In doing so, the payer is seeking to compensate physicians for the time spent explaining to patients with a viral diagnosis why antibiotics are not indicated. The provider decides whether to prescribe antibiotics to patients they have diagnosed with viral infection. We consider both interpretation of symptom presentation and technical diagnostic testing for diagnostic accuracy and so we explore the possibility that incentive payments may affect the diagnosis behavior of the provider.

Our research provides practical guidance for payers regarding incentive payments to manage antibiotic use and reduce the frequency of inappropriate prescriptions. Objective diagnostic information is not available in all circumstances and so diagnostic classification relies on physician assessment of clinical symptoms. In this case, diagnostic accuracy becomes endogenous and it is possible that incentive payments will manipulate a provider’s diagnostic assessments. In this setting, we show that a simple action-based payment contract with bonus payment can coordinate provider behavior to socially optimal antibiotic prescription levels. We demonstrate that the bonus payments establish a prescription threshold within the symptom distribution domain. According to this threshold policy, only patients with symptoms above this threshold should be diagnosed with bacterial infection and prescribed antibiotics. Further, we show that, in a scenario where diagnosis relies on additional technical point-of-care testing, there is a socially optimal antibiotic prescription threshold along the symptom distribution domain. Consequently, patients diagnosed with a viral infection who exhibit symptom levels below this threshold will not receive antibiotics. Importantly, the rate of inappropriate antibiotic prescriptions is reduced when diagnoses are based on an additional technical diagnostics. Our results show that adding a bonus payment to the contract terms of providers can only co-ordinate the system when the opportunity cost of the provider is public information.

In realistic situations, a provider’s opportunity cost is private information and a bonus payment creates a moral hazard where, in an effort to increase revenue from the bonus, the provider changes their prescription threshold decreasing the proportion of patients receiving antibiotics. Providers with lower opportunity cost may benefit from information rent by not prescribing antibiotics including to patients that they would have otherwise diagnosed with a bacterial infection, but now diagnose as having a viral infection in order to obtain the bonus. Therefore, while incentive payments would reduce the frequency of inappropriate antibiotic prescriptions, when compared to the first-best policy, providers with high opportunity cost would continue to over-prescribe antibiotics even with bonus payments and providers with a low opportunity cost may over-respond to the incentives adversely affecting patient care. Through extensive numerical studies, we show that when opportunity cost is private information, a menu of contracts does not significantly improve the outcome achieved with a single contract.

Our analysis focuses on one factor influencing inappropriate antibiotic prescribing behavior (avoiding opportunity cost) and one type of stewardship effort (pay-for-performance contracts). Inappropriate prescribing practices include selecting the wrong antibiotic, selection of a broad-spectrum antibiotic when a narrow-spectrum antibiotic would suffice, or prescribing antibiotics for the wrong duration. Opportunity cost is also not the only reason that providers prescribe antibiotics inappropriately. Providers may also be uncertain about which symptom combinations are clinically indicated for antibiotics under the most recent clinical guidelines, the frequency of side effects from antibiotics, or the social harm of inappropriate antibiotic prescriptions. An action-based contract will be most effective if partnered with other antibiotic stewardship efforts demonstrated to reduce inappropriate antibiotic prescribing.

That said, our model provides insight into the effectiveness of other types of stewardship programs. Many programs seek to reduce the opportunity costs for providers by educating patients about when to realistically expect antibiotics and educating physicians about how to engage in effective patient communication. Other stewardship programs target physicians’ diagnostic skills, reducing physicians’ fears of misdiagnosis, and providing physicians access to rapid on-site diagnostics. The availability of point-of-care diagnostic testing can transition some clinical assessments from relying on the interpretation of symptoms to an exogenous diagnostic setting, while increasing physician confidence in the diagnosis. This is important because we found that bonus payments are more effective at reducing inappropriate antibiotic prescribing when diagnostic tests have high specificity. Therefore, clinical diagnostic decisions in which point-of-care diagnostics are available to differentiate between viral and bacterial infections are potentially ideal settings in which the addition of a bonus payment can reduce the rate of inappropriate antibiotic prescribing. However, it is notable that even in these settings, diagnostic accuracy is not strictly exogenous as the provider must also make the decision to use the diagnostic test. While we have shown that it is possible to achieve the socially optimal use of antibiotics using a bonus without risk of manipulating patient diagnoses in settings in which there is a technical diagnostic, the question of whether it is valuable to add a technical diagnostic or incentivize the use of a technical diagnostic depends on features of that test including its cost.

## Supplemental Material

sj-pdf-1-pao-10.1177_10591478241264022 - Supplemental material for Influencing Primary Care Antibiotic Prescription Behavior Using Financial Incentives Supplemental material, sj-pdf-1-pao-10.1177_10591478241264022 for Influencing Primary Care Antibiotic Prescription Behavior Using Financial Incentives by Salar Ghamat, Mojtaba Araghi, Lauren E Cipriano and Michael Silverman in Production and Operations Management
