# Supplementary material for: Influencing Primary Care Antibiotic Prescription Behavior Using Financial Incentives
Source: Prod Oper Manag. 2024 Jul 26;33(10):2051–72. doi: 10.1177/10591478241264022 (PMC13021006; doi:10.1177/10591478241264022)
Supplement: sj-pdf-1-pao-10.1177_10591478241264022 - Supplemental material for Influencing Primary Care Antibiotic Prescription Behavior Using Financial Incentives [file sj-pdf-1-pao-10.1177_10591478241264022.pdf]

E-Companion A – Proofs

Proof of Lemma 1: Given a patient with symptom s diagnosed with a viral infection, the provider should decide whether to prescribe antibiotics $d(s) = 1$ or not $d(s) = 0$. Following the paper's notation, we define conditional probabilities of True Viral and False Viral for a patient with symptom s:

$$\text{P(TV}(s))\text{=P(Viral infection} \mid \text{Viral diagnosis)}$$

$$\text{P(FV}(s))\text{=P(Bacterial infection} \mid \text{Viral diagnosis)}$$

Let Π_P^j represent the provider's payoff under Outcome $j \in \{A, B, C, D\}$ which includes their altruistic utility from the health outcome of their patients, the corresponding payments received from the payer, and their opportunity cost. Similarly, we can denote the central planer's payoff of each outcome by Π_C^j.

It follows from the decision tree in Figure 1 that the expected payoff of prescribing antibiotics is:

$$\Pi_j\big(d(s)=1\big) = P\big(\text{FV}(s)\big)\,\Pi_j^A + P\big(\text{TV}(s)\big)\,\Pi_j^B, \qquad j \in \{P, C\}$$

and the expected payoff of not prescribing antibiotics is:

$$\Pi_j\big(d(s)=0\big) = P\big(\text{FV}(s)\big)\,\Pi_j^C + P\big(\text{TV}(s)\big)\,\Pi_j^D, \qquad j \in \{P, C\}$$

Therefore the marginal payoff of prescribing antibiotics to a patient with symptom s diagnosed with a viral infection is:

$$\Pi_j\big(d(s)=1\big) - \Pi_j\big(d(s)=0\big) = P\big(\text{FV}(s)\big)\left(\Pi_j^A - \Pi_j^C\right) + P(\text{TV}(s))\left(\Pi_j^B - \Pi_j^D\right), \qquad j \in \{P, C\} \quad \text{(A1)}$$

To prove the optimality of the threshold policy, it is enough to show that the marginal payoff is monotone increasing in s. Intuitively, this property holds as (i) the likelihood of a false viral diagnosis increases with s; (ii) it is beneficial to prescribe antibiotics to a patient with bacterial infection (Outcome A) as opposed to not prescribing them (Outcome C); and (iii) it is harmful to prescribe antibiotics to a patients with a viral infection (Outcome B) as opposed to not prescribing them (Outcome D).

In each of the corresponding proofs (i.e., for both the integrated and decenteralized care systems, and both when diagnosis relies on symptom presentation or on additional testing), we explicitly show that the marginal payoff is monotone increasing in s and thus the threshold policy is optimal. □

Proof of Proposition 1: Substituting $P\big(FV(s)\big) = p(s)$, $\Pi_C^A - \Pi_C^C = (1+\alpha)B(1-\delta) + k(s)$, and $\Pi_C^B - \Pi_C^D = -((1+\alpha)F + c + h) + k(s)$ in Equation (A1), the marginal profit of prescribing antibiotics to a patient with symptom s diagnosed with a viral infection becomes

$$p(s)\big((1+\alpha)B(1-\delta)\big) - (1-p(s))\big((1+\alpha)F + c + h\big) + k(s),$$

which is monotone increasing in s as both $p(s)$ and $k(s)$ are increasing in s. Therefore, by Lemma 1, the central planer's optimal prescription follows a threshold policy.

Substituting Equation (3) in Equation (4), and setting $\theta = \tau$, yields

$$\begin{aligned}\Pi_C(\tau, B, F, c, h, k) = &\int_\tau^1 p(s) f_s(s)[U_S(B,F) + U_P(B,F) - c]\,\mathrm{d}s \\ &+ \int_\tau^1 (1-p(s)) f_s(s)[U_S(0,F) + U_P(0,F) - c - h]\,\mathrm{d}s \\ &+ \int_0^\tau p(s) f_s(s)[U_S(\delta B, F) + U_P(\delta B, F) - k(s) - c]\,\mathrm{d}s \\ &+ \int_0^\tau (1-p(s)) f_s(s)[U_S(0,0) + U_P(0,0) - k(s)]\,\mathrm{d}s\end{aligned} \tag{A2}$$

From (A2), we have

$$\begin{aligned}\frac{\partial^2 \Pi_C}{\partial \tau^2} = &f_s'(\tau)(c + \alpha F + F + h - k(\tau) - p(\tau)((1+\alpha)(B - \delta B) + c + \alpha F + F + h)) \\ &- f_s(\tau)(k'(\tau) + p'(\tau)\,((1+\alpha)(B - \delta B) + c + \alpha F + F + h))\end{aligned}$$

We have $\frac{\partial^2 \Pi_C}{\partial \tau^2} < 0$ if (i) $f_s'(\tau) < 0$ and $k(\tau) < \bar{\bar{k}}^{\mathrm{P}}(\tau)$, where

$$\begin{aligned}\bar{\bar{k}}^{\mathrm{P}}(\tau) = -\frac{1}{f_s'(\tau)}\Big(&f_s(\tau)(p'(\tau)(B(1+\alpha)(1-\delta) + (1+\alpha)F + c + h) + k'(\tau)) \\ &+ f_s'(\tau)(p(\tau)(B(1+\alpha)(1-\delta) + h) - (1-p)(c + (1+\alpha)F) - h)\Big)\end{aligned}$$

or (ii) $f_s'(\tau) > 0$ and $k(\tau) > \bar{\bar{k}}^{\mathrm{P}}(\tau)$ or (iii) $f_s'(\tau) = 0$. The inequality follows from the fact that $p(\tau)$ and $k(\tau)$ are increasing in τ. We have $\frac{\partial \Pi_C}{\partial \tau} = f_s(\tau)(c + h - k(\tau) + F(1+\alpha) - (c + h + B(1+\alpha)(1-\delta) + F(1+\alpha))p(\tau))$. Thus $\frac{\partial \Pi_C}{\partial \tau} < 0 \Leftrightarrow k(\tau) > (1-p(\tau))\Big((1+\alpha)F + c + h\Big) - p(\tau)\Big((1+\alpha)B(1-\delta)\Big) \equiv \hat{k}^{\mathrm{P}}(\tau)$. Further, $\frac{\hat{k}^{\mathrm{P}}(\tau)}{\partial \tau} < 0$. If $k(0) > \hat{k}^{\mathrm{P}}(0)$, the largest opportunity cost threshold, then $\frac{\partial \Pi_C}{\partial \tau} < 0$, thus $\tau^* = 0$. To eliminate the unrealistic cases, in the paper, we assume that the smallest opportunity cost threshold $\hat{k}^{\mathrm{P}}(1) \le 0 \le k(1)$; however, if this is not the case, if $k(1) < \hat{k}^{\mathrm{P}}(1)$, then $\frac{\partial \Pi_C}{\partial \tau} > 0$, thus $\tau^* = 1$. On the other hand, when $f_s'(\tau) \le 0$, we have $\hat{k}^{\mathrm{P}}(\tau) < \bar{\bar{k}}^{\mathrm{P}}(\tau)$ and when when $f_s'(\tau) > 0$, we have $\hat{k}^{\mathrm{P}}(\tau) > \bar{\bar{k}}^{\mathrm{P}}(\tau)$. Therefore, when $\hat{k}^{\mathrm{P}}(1) \le k(\tau) \le \hat{k}^{\mathrm{P}}(0)$, τ^* satisfies $\frac{\partial \Pi_C}{\partial \tau} = 0$ because $\frac{\partial^2 \Pi_C}{\partial \tau^2} < 0$ at the critical point. Thus, we have:

$$\tau^* = \arg\left\{0 \le \tau \le 1 : p(\tau) = \frac{(1+\alpha)F + c + h - k(\tau)}{(1+\alpha)F + c + h + (1+\alpha)B(1-\delta)}\right\}.\Box$$

Proof of Lemma 2: Substituting $P\big(FV(s)\big) = p(s)$, $\Pi_P^A - \Pi_P^C = \alpha B(1-\delta) + k(s) - w - b$, and $\Pi_P^B - \Pi_P^D = -\alpha F - b + k(s)$ in Equation (A1), the marginal profit of prescribing antibiotics to a patient with symptom s diagnosed with a viral infection becomes

$$p(s)\left(\alpha B(1-\delta) - w\right) - (1-p(s))\,(\alpha F) + k(s) - b,$$

which is monotone increasing in s as both $p(s)$ and $k(s)$ are increasing in s (see Remark A1). Therefore, by Lemma 1, the provider's optimal prescription follows a threshold policy.

Remark A1: If the offered visit fee w exceeds the cost associated with delayed care $\alpha B(1-\delta)$, the marginal profit of prescribing antibiotics may become non-monotone. This is an unrealistic case in which a provider does not prescribe antibiotics to a patient with bacterial infection, hoping to receive a second visit fee. However, we show at the end of the proof, in such cases, the optimal prescription decision will be a corner point of the symptom range. Also, we show in E-companion C-1 that our results hold in the absence of the second visit fee.

Substituting Equation (3) in Equation (1), and setting $\theta = \tau$, yields

$$\begin{aligned}\Pi_P(\tau, B, F, k, \zeta) = &\int_\tau^1 p(s) f_s(s)\,[U_P(B,F) + w]\;\mathrm{d}s \\ &+ \int_\tau^1 (1-p(s)) f_s(s)\,[U_P(0,F) + w]\;\mathrm{d}s \\ &+ \int_0^\tau p(s) f_s(s)\,[U_P(\delta B, F) + 2w + b - k(s)]\;\mathrm{d}s \\ &+ \int_0^\tau (1-p(s)) f_s(s)\,[U_P(0,0) + w + b - k(s)]\;\mathrm{d}s\end{aligned} \tag{A3}$$

From (A3), we have, $\frac{\partial^2 \Pi_P}{\partial \tau^2} = (b - k(\tau) + \alpha F + (w - \alpha(B - \delta B + F))p(\tau))\; f_s'(\tau) + f_s(\tau)(p'(\tau)(w - \alpha(B - \delta B + F)) - k'(\tau))$ and $\frac{\partial \Pi_P}{\partial \tau} = (b - k(\tau) + \alpha F + (w - \alpha(B - \delta B + F))p(\tau)) f_s(\tau)$. We can show that $\frac{\partial \Pi_P}{\partial \tau} < 0 \Leftrightarrow b < -(1-p(\tau))(\alpha F - k(\tau)) + p(\tau)\Big(\alpha B\,(1-\delta) + k(\tau) - w\Big) = \underline{b}^{\mathrm{P}}(\tau)$ – minimum bonus required for the provider not to prescribe antibiotics to a patient with symptom level τ. Further, we have $\frac{\partial \Pi_P}{\partial \tau} = 0 \to \tau^*(w,b) = \arg\Big\{0 \le \tau \le 1 : p(\tau) = \frac{\alpha F - k(\tau) + b}{\alpha(B - \delta B + F) - w}\Big\}$ and at this critical point, $\frac{\partial^2 \Pi_P}{\partial \tau^2}|_{\tau^*(w,b)} = f_s(\tau^*(w,b))\big((w - \alpha(B - \delta B + F))p'(\tau^*(w,b)) - k'(\tau^*(w,b))\big)$ which is negative if $w < \frac{k'(\tau^*(w,b))}{p'(\tau^*(w,b))} + \alpha(B - \delta B + F)$. Under such condition, if $b < \underline{b}^{\mathrm{P}}(0)$, then $\frac{\partial \Pi_P}{\partial \tau} < 0$, thus $\tau^*(w,b) = 0$. If $b > \underline{b}^{\mathrm{P}}(1)$, then $\frac{\partial \Pi_P}{\partial \tau} > 0$, thus $\tau^*(w,b) = 1$.

If $w > \frac{k'(\tau^*(w,b))}{p'(\tau^*(w,b))} + \alpha(B - \delta B + F)$, we have $\frac{\partial^2 \Pi_P}{\partial \tau^2} > 0$, which means $\tau^* = 0$ if $\Pi_P(\mathcal{P}(0), B, F, k(0), \zeta) > \Pi_P(\mathcal{P}(1), B, F, k(1), \zeta) \to b \le (\alpha\,(B - \delta B - F) - w)\,\mathbb{E}(p) + \mathbb{E}(k) - \alpha F$ where $\mathbb{E}(k) = \int_0^1 k(s) f_s(s)\,\mathrm{d}s$ and $\mathbb{E}(p) = \int_0^1 p(s) f_s(s)\,\mathrm{d}s$; and $\tau^* = 1$ otherwise. $\square$

Proof of Proposition 2: Substituting Equation (3) in Equation (2), and setting $\theta=\tau$, yields

$$\begin{aligned}\Pi_S(\tau,B,F,c,h,\zeta)=&\int_{\tau}^{1} p(s)f_s(s)\left[U_S(B,F)-w-c\right]\mathrm{d}s\\&+\int_{\tau}^{1}(1-p(s))f_s(s)\left[U_S(0,F)-w-c-h\right]\mathrm{d}s\\&+\int_{0}^{\tau}p(s)f_s(s)\left[U_S(\delta B,F)-2w-b-c\right]\mathrm{d}s\\&+\int_{0}^{\tau}(1-p(s))f_s(s)\left[U_S(0,0)-w-b\right]\mathrm{d}s\end{aligned}\tag{A4}$$

From (A4), we have, $\frac{\partial \Pi_S}{\partial \tau}=\big((1-p(s))(F+c+h-b)-p(s)(b+B\,(1-\delta)+w)\big)f_s(\tau)$. Knowing the provider's best response, the payer would provide the minimum bonus $\underline{b}^{\mathrm{P}}(\tau)$ to prevent antibiotic prescription to a patient with symptom level τ if and only if $\frac{\partial \Pi_S}{\partial \tau}\geq 0 \Leftrightarrow \big(1-p(s)\big)\big(F+c+h-\underline{b}^{\mathrm{P}}(\tau)\big)-p(s)\big(\underline{b}^{\mathrm{P}}(\tau)+B\,(1-\delta)+w\big)\geq 0 \Leftrightarrow k(\tau)\leq \hat{k}^{\mathrm{P}}(\tau)$.

Solving OPT1, we have $\frac{\partial^2 \Pi_S}{\partial w^2}=0$ and $\frac{\partial \Pi_S}{\partial w}=-\left(\int_0^1 f_s(s)\,\mathrm{d}s+\int_0^{\tau}p(s)f_s(s)\,\mathrm{d}s\right)<0$. Therefore, regardless of the antibiotic prescription behaviour of the provider, the payer sets w such that it absorbs the surplus of the provider (binding individual-rationality constraint in OPT1). We know that, when $k(\tau)>\hat{k}^{\mathrm{P}}(\tau)$ the payer will not offer the minimum bonus required by the provider not to prescribe antibiotics to a patient with symptom level τ (i.e., $b<\underline{b}^{\mathrm{P}}(\tau)$). Therefore, we have the following.

1. When $k(0)>\hat{k}^{\mathrm{P}}(0)\rightarrow b<\underline{b}^{\mathrm{P}}(0)$, and thus $\tau^*=0$. In this case, $\Pi_P|_{b<\underline{b}^{\mathrm{P}}(0);\tau^*=0}=\underline{\Pi}_P \Leftrightarrow w=\underline{\Pi}_P+\alpha(F-B\mathbb{E}(p))$.

2. When $\hat{k}^{\mathrm{P}}(1)\leq k(\tau)\leq \hat{k}^{\mathrm{P}}(0)$, since $\frac{\partial \hat{k}^{\mathrm{P}}(\tau)}{\partial \tau}<0$, the payer will offer the minimum bonus required by the provider (i.e., $b=\underline{b}^{\mathrm{P}}(\tau^*)$) up to a patient with symptom level τ^* such that $k(\tau^*(w,b))=\hat{k}^{\mathrm{P}}(\tau^*(w,b))$ which yields $b=\frac{k(\tau)(B(1-\delta)+c+F+h+w)-wF(1+\alpha)-(c+h)(w-\alpha B(1-\delta))}{(1+\alpha)F+c+h+(1+\alpha)B(1-\delta)}$. Replacing b in $\tau^*(w,b)$ we get $\tau^*=\arg\left\{0\leq\tau\leq 1: p(\tau)=\frac{(1+\alpha)F+c+h-k(\tau)}{(1+\alpha)F+c+h+(1+\alpha)B(1-\delta)}\right\}$. In this case, $\Pi_P|_{b=\underline{b}^{\mathrm{P}}(\tau^*);\tau^*}=\underline{\Pi}_P \Leftrightarrow$

$$\begin{aligned}w=&\Bigg(\alpha(B(1-\delta)+F)(c+(1+\alpha)F+h)\int_0^{\tau^*}f_s(s)ds-\alpha(B(1-\delta)+F)\int_0^{\tau^*}f_s(s)k(s)ds\\&-\Psi(\underline{\Pi}_P-\alpha(B\mathbb{E}(p)-F-(F+B(1-\delta))\int_0^{\tau^*}f_s(s)p(s)ds))\Bigg)\\&*\left((c+(1+\alpha)F+h)\int_0^{\tau^*}f_s(s)ds-\int_0^{\tau^*}f_s(s)k(s)ds-\Psi(1+\int_0^{\tau^*}f_s(s)p(s)ds)\right)^{-1}\end{aligned}$$

where $\Psi=(1+\alpha)F+c+h+(1+\alpha)B(1-\delta)$.

3. In the paper, we assume that $\hat{k}^{\mathrm{P}}(1)\leq 0\leq k(1)$; however, if this is not the case, when $k(1)<\hat{k}^{\mathrm{P}}(1)\rightarrow b>\underline{b}^{\mathrm{P}}(1)$, and thus $\tau^*=1$. In this case, $\Pi_P|_{b=\underline{b}^{\mathrm{P}}(1);\tau^*=1}=\underline{\Pi}_P \Leftrightarrow w=\alpha(B(1-\delta)+F)+\frac{\underline{\Pi}_P+\mathbb{E}(k)-\alpha B(\mathbb{E}(p)+1-\delta)-k(1)}{1+\mathbb{E}(p)-p(1)}$. □

Proof of Corollary 1: When the payer offers $\zeta=\{w\}$, the provider and the payer's payoff functions (Equations 1-2 in the main text) will change to the following

$$\begin{aligned}\Pi_P|_{\zeta=\{w\}} &= \int_\tau^1 p(s)f_s(s)\left[U_P(B,F)+w\right]\mathrm{d}s + \int_\tau^1 (1-p(s))f_s(s)\left[U_P(0,F)+w\right]\mathrm{d}s \\ &+ \int_0^\tau p(s)f_s(s)\left[U_P(\delta B,F)+2w-k(s)\right]\mathrm{d}s + \int_0^\tau (1-p(s))f_s(s)\left[U_P(0,0)+w-k(s)\right]\mathrm{d}s \\ \Pi_S|_{\zeta=\{w\}} &= \int_\tau^1 p(s)f_s(s)\left[U_S(B,F)-w-c\right]\mathrm{d}s + \int_\tau^1 (1-p(s))f_s(s)\left[U_S(0,F)-w-c-h\right]\mathrm{d}s \\ &+ \int_0^\tau p(s)f_s(s)\left[U_S(\delta B,F)-2w-c\right]\mathrm{d}s + \int_0^\tau (1-p(s))f_s(s)\left[U_S(0,0)-w\right]\mathrm{d}s \end{aligned} \tag{A5}$$

For the provider, we have, $\frac{\partial^2\Pi_P|_{\zeta=\{w\}}}{\partial\tau^2} = f_s'(\tau)\left(p(\tau)(\alpha\delta B-\alpha(B+F)+w)+\alpha F-k(\tau)\right) + f_s(\tau)\left(p'(\tau)(\alpha\delta B-\alpha(B+F)+w)-k'(\tau)\right)$ and $\frac{\partial\Pi_P|_{\zeta=\{w\}}}{\partial\tau} = (-k(\tau)+\alpha F+(w-\alpha(B-\delta B+F))p(\tau))f_s(\tau)$. We can show that $\frac{\partial\Pi_P|_{\zeta=\{w\}}}{\partial\tau}<0 \Leftrightarrow w<\frac{k(\tau)-\alpha F+\alpha(F+B-\delta B)p(\tau)}{p(\tau)} = \underline{w}^{\mathrm{P}}(\tau)$ – minimum visit fee required for the provider not to prescribe antibiotics to a patient with symptom level τ. Further, we have $\frac{\partial\Pi_P|_{\zeta=\{w\}}}{\partial\tau}=0 \to \tau^*(w)=\arg\left\{0\le\tau\le1 : p(\tau)=\frac{\alpha F-k(\tau)}{\alpha(B-\delta B+F)-w}\right\}$ and at this critical point, $\frac{\partial^2\Pi_P|_{\zeta=\{w\}}}{\partial\tau^2}|_{\tau^*(w)} = f_s(\tau^*(w))\big((w-\alpha(B-\delta B+F))p'(\tau^*(w))-k'(\tau^*(w))\big)$ which is negative if $w<\frac{k'(\tau^*(w))}{p'(\tau^*(w))}+\alpha(B-\delta B+F)$. Under such condition, if $w<\underline{w}^{\mathrm{P}}(0)$, then $\frac{\partial\Pi_P|_{\zeta=\{w\}}}{\partial\tau}<0$, thus $\tau^*(w)=0$. If $w>\underline{w}(1)$, then $\frac{\partial\Pi_P|_{\zeta=\{w\}}}{\partial\tau}>0$, thus $\tau^*(w)=1$.

On the other hand, for the payer, we have $\frac{\partial\Pi_S|_{\zeta=\{w\}}}{\partial\tau} = \big((1-p(s))(F+c+h)-p(s)(B(1-\delta)+w)\big)f_s(\tau)$. Knowing the provider's best response, the payer *may* provide the minimum visit $\underline{w}^{\mathrm{P}}(\tau)$ to prevent antibiotic prescription to a patient with symptom level τ if and only if $\frac{\partial\Pi_S|_{\zeta=\{w\}}}{\partial\tau}\ge0 \Leftrightarrow \big(1-p(s)\big)\big(F+c+h\big)-p(s)\big(B(1-\delta)+\underline{w}(\tau)\big)\ge0 \Leftrightarrow k(\tau)\le\hat{k}^{\mathrm{P}}(\tau)$.

However, unlike in the $\zeta=\{w,b\}$ contract, with $\zeta=\{w\}$ the payer may not always want to disincentivize antibiotic prescription when $k(\tau)\le\hat{k}^{\mathrm{P}}(\tau)$. This is because, with $\zeta=\{w\}$, the payer cannot absorb the provider's surplus and achieve optimal antibiotic prescription behaviour simultaneously. We have $\frac{\partial^2\Pi_S|_{\zeta=\{w\}}}{\partial w^2}=0$ and $\frac{\partial\Pi_S|_{\zeta=\{w\}}}{\partial w} = -\left(\int_0^1 f_s(s)\,\mathrm{d}s+\int_0^\tau p(s)f_s(s)\,\mathrm{d}s\right)<0$; therefore, instead of disincentivizing antibiotic prescription, the payer can offer the lowest possible visit fee by binding the individual rationality constraint in OPT1: $\Pi_P(\tau(w))=\underline{\Pi}_P \Leftrightarrow w = \frac{\underline{\Pi}_P+\alpha F-\alpha F\int_0^\tau f_s(s)ds+\int_0^\tau f_s(s)k(s)ds-\alpha B\mathbb{E}(p)+\alpha(F+B(1-\delta))\int_0^\tau p(s)f_s(s)ds)}{1+\int_0^\tau p(s)f_s(s)ds} = \underline{\underline{w}}^{\mathrm{P}}$. When $k(\tau)\le\hat{k}^{\mathrm{P}}(\tau)$ we have: $\underline{\underline{w}}^{\mathrm{P}}\ne\underline{w}^{\mathrm{P}}$. We can show that $\exists B,F,c,h\in\mathbb{R}_{\ge0} : \Pi_S|_{w=\underline{w}^{\mathrm{P}};\tau^*}\le\Pi_S|_{w=\underline{\underline{w}}^{\mathrm{P}};\tau}$. Therefore, with $\zeta=\{w\}$ the payer does not prefer to achieve the socially optimal antibiotic prescription levels. □

Proof of Proposition 3: Substituting $P\big(FV(s)\big) = \frac{p(s)(1-q_1)}{(1-p(s))q_2+p(s)(1-q_1)}$, $\Pi_C^A - \Pi_C^C = (1+\alpha)B(1-\delta) + k(s)$, and $\Pi_C^B - \Pi_C^D = -((1+\alpha)F + c + h) + k(s)$ in Equation (A1), the marginal profit of prescribing antibiotics to a patient with symptom s diagnosed with a viral infection becomes

$$\frac{p(s)(1-q_1)}{(1-p(s))q_2+p(s)(1-q_1)}\big((1+\alpha)B(1-\delta)\big) - \left(1 - \frac{p(s)(1-q_1)}{(1-p(s))q_2+p(s)(1-q_1)}\right)\big((1+\alpha)F+c+h\big)+k(s),$$

which is monotone increasing in s as both $\frac{p(s)(1-q_1)}{(1-p(s))q_2+p(s)(1-q_1)}$ and $k(s)$ are increasing in s. Therefore, by Lemma 1, the central planer's optimal prescription follows a threshold policy.

Substituting health outcome probabilities from Equation (9) in Equation (4) we have

$$\begin{aligned}\Pi_C(\tau,B,F,c,h,k) = &\left(\int_0^1 q_1 p(s) f_s(s)\,\mathrm{d}s + \int_\tau^1 (1-q_1)p(s)f_s(s)\,\mathrm{d}s\right)[U_S(B,F)+U_P(B,F)-c]\\ &+\left(\int_0^1 (1-q_2)(1-p(s))f_s(s)\,\mathrm{d}s + \int_\tau^1 q_2(1-p(s))f_s(s)\,\mathrm{d}s\right)[U_S(0,F)+U_P(0,F)-c-h]\\ &+\left(\int_0^\tau (1-q_1)p(s)f_s(s)\,\mathrm{d}s\right)[U_S(\delta B,F)+U_P(\delta B,F)-k(s)-c]\\ &+\left(\int_0^\tau q_2(1-p(s))f_s(s)\,\mathrm{d}s\right)[U_S(0,0)+U_P(0,0)-k(s)]\end{aligned} \tag{A6}$$

From (A6), we have

$$\begin{aligned}\frac{\partial^2 \Pi_C}{\partial \tau^2} = &f_s'(\tau)\Big(q_2(1-p(\tau))(c+\alpha F+F+h-k(\tau)) - (1-q_1)p(\tau)((1+\alpha)(B-\delta B)+k(\tau))\Big)\\ &- f_s(\tau)\Big((1-q_1)(p'(\tau)((1+\alpha)(B-\delta B)+k(\tau))+p(\tau)k'(\tau))\\ &+ q_2\left(p'(\tau)(c+\alpha F+F+h-k(\tau))+(1-p(\tau))k'(\tau)\right)\Big)\end{aligned}$$

We have $\frac{\partial^2 \Pi_C}{\partial \tau^2} < 0$ if (i) $f_s'(\tau) < -\frac{f_s(\tau)p'(\tau)(q_1+q_2-1)}{p(\tau)(q_1+q_2-1)-q_2}$ and $k(\tau) < \bar{\bar{k}}^{\mathrm{T}}(\tau)$, where

$$\begin{aligned}\bar{\bar{k}}^{\mathrm{T}}(\tau) = &\frac{1}{(q_1+q_2-1)(f_s(\tau)p'(\tau)+f_s'(\tau)p(\tau)) - f_s'(\tau)q_2}\Big((\alpha+1)B(\delta-1)(q_1-1)(f_s(\tau)p'(\tau)+f_s'(\tau)p(\tau))\\ &+ f_s(\tau)p'(\tau)q_2(c+\alpha F+F+h) + f_s'(\tau)(p-1)q_2(c+\alpha F+F+h) + f_s(\tau)k'(\tau)(q_2-p(q_1+q_2-1)\Big)\end{aligned}$$

or (ii) $f_s'(\tau) > -\frac{f_s(\tau)p'(\tau)(q_1+q_2-1)}{p(\tau)(q_1+q_2-1)-q_2}$ and $k(\tau) > \bar{\bar{k}}^{\mathrm{T}}(\tau)$ or (iii) $f_s'(\tau) = -\frac{f_s(\tau)p'(\tau)(q_1+q_2-1)}{p(\tau)(q_1+q_2-1)-q_2}$. The inequality follows from the fact that $p(\tau)$ and $k(\tau)$ are increasing in τ. We have $\frac{\partial \Pi_C}{\partial \tau} = f_s(\tau)(q_2((1-p(\tau))(c-k(\tau)+\alpha F+F+h)) - (1-q_1)p(\tau)((\alpha+1)B(1-\delta)+k(\tau)))$. Thus $\frac{\partial \Pi_C}{\partial \tau} < 0 \Leftrightarrow k(\tau) > \frac{(1-p(\tau))q_2((1+\alpha)F+c+h)-p(\tau)(1-q_1)(1+\alpha)B(1-\delta)}{p(\tau)(1-q_1)+(1-p(\tau))q_2} \equiv \hat{k}^{\mathrm{T}}(\tau)$.

Further, $\frac{\hat{k}^{\mathrm{T}}(\tau)}{\partial \tau} < 0$. If $k(0) > \hat{k}^{\mathrm{T}}(\tau)$, the largest opportunity cost threshold, then $\frac{\partial \Pi_C}{\partial \tau} < 0$, thus $\tau^* = 0$. To eliminate the unrealistic cases, in the paper, we assume that the smallest opportunity cost threshold $\hat{k}^{\mathrm{T}}(1) \le 0 \le k(1)$; however, if this is not the case, if $k(1) < \hat{k}^{\mathrm{T}}(1)$, then $\frac{\partial \Pi_C}{\partial \tau} > 0$, thus $\tau^* = 1$. On the other hand, when $f_s'(\tau) \le -\frac{f_s(\tau)p'(\tau)(q_1+q_2-1)}{p(\tau)(q_1+q_2-1)-q_2}$, we have $\hat{k}^{\mathrm{T}}(\tau) < \bar{\bar{k}}^{\mathrm{T}}(\tau)$ and when

when $f_s'(\tau) > -\frac{f_s(\tau)p'(\tau)(q_1+q_2-1)}{p(\tau)(q_1+q_2-1)-q_2}$, we have $\hat{k}^{\mathrm{T}}(\tau) > \bar{\bar{k}}^{\mathrm{T}}(\tau)$. Therefore, when $\hat{k}^{\mathrm{T}}(1) \le k(\tau) \le \hat{k}^{\mathrm{T}}(0)$, τ^* satisfies $\frac{\partial \Pi_C}{\partial \tau} = 0$ because $\frac{\partial^2 \Pi_C}{\partial \tau^2} < 0$ at the critical point. Thus, we have:

$$\tau^* = \arg\left\{0 \le \tau \le 1 : p(\tau) = \frac{q_2\left((1+\alpha)F + c + h - k(\tau)\right)}{q_2\left((1+\alpha)F + c + h - k(\tau)\right) + (1-q_1)\left((1+\alpha)B(1-\delta) + k(\tau)\right)}\right\}. \square$$

Proof of Lemma 3: Substituting $P\big(FV(s)\big) = \frac{p(s)(1-q_1)}{(1-p(s))q_2 + p(s)(1-q_1)}$, $\Pi_P^A - \Pi_P^C = \alpha B(1-\delta) + k(s) - w - b$, and $\Pi_P^B - \Pi_P^D = -\alpha F - b + k(s)$ in Equation (A1), the marginal profit of prescribing antibiotics to a patient with symptom s diagnosed with a viral infection becomes

$$\frac{p(s)(1-q_1)}{(1-p(s))q_2 + p(s)(1-q_1)}\left(\alpha B(1-\delta) - w\right) - \left(1 - \frac{p(s)(1-q_1)}{(1-p(s))q_2 + p(s)(1-q_1)}\right)\left(\alpha F\right) + k(s) - b,$$

which is monotone increasing in s as both $\frac{p(s)(1-q_1)}{(1-p(s))q_2+p(s)(1-q_1)}$ and $k(s)$ are increasing in s (see Remark A1 in the Proof of Lemma 2). Therefore, by Lemma 1, the provider's optimal prescription follows a threshold policy.

Substituting Equation (9) in Equation (1) we have

$$\begin{aligned}\Pi_P(\tau, B, F, k, \zeta) = &\left(\int_0^1 q_1 p(s) f_s(s)\,\mathrm{d}s + \int_\tau^1 (1-q_1)p(s)f_s(s)\,\mathrm{d}s\right)[U_P(B,F) + w] \\ &+ \left(\int_0^1 (1-q_2)(1-p(s))f_s(s)\,\mathrm{d}s + \int_\tau^1 q_2(1-p(s))f_s(s)\,\mathrm{d}s\right)[U_P(0,F) + w] \\ &+ \int_0^\tau (1-q_1)p(s)f_s(s)[U_P(\delta B, F) + 2w + b - k(s)]\,\mathrm{d}s \\ &+ \int_0^\tau q_2(1-p(s))f_s(s)[U_P(0,0) + w + b - k(s)]\,\mathrm{d}s\end{aligned} \tag{A7}$$

From (A7), we have,

$$\begin{aligned}\frac{\partial^2 \Pi_P}{\partial \tau^2} = &f_s'(\tau)\big((1-q_1)p(\tau)(b - \alpha B(1-\delta) - k(\tau) + w) + q_2(1-p(\tau))(b - k(\tau) + \alpha F)\big) \\ &- f_s(\tau)\big((1-q_1)\left(p(\tau)k'(\tau) - p'(\tau)(b - \alpha B(1-\delta) - k(\tau) + w)\right) \\ &+ q_2\left(p'(\tau)(b - k(\tau) + \alpha F) + (1-p(\tau))k'(\tau)\right)\big)\end{aligned}$$

and $\frac{\partial \Pi_P}{\partial \tau} = ((1-q_1)p(\tau)(b - \alpha B(1-\delta) - k(\tau) + w) + q_2((1-p(\tau))(b - k(\tau) + \alpha F)))f_s(\tau)$. We can show that $\frac{\partial \Pi_P}{\partial \tau} < 0 \Leftrightarrow b < \frac{-(1-p(\tau))q_2(\alpha F - k(\tau)) + p(\tau)(1-q_1)(\alpha B(1-\delta) + k(\tau) - w)}{p(\tau)(1-q_1) + (1-p(\tau))q_2} = \underline{b}^{\mathrm{T}}(\tau)$ – minimum bonus required for the provider not to prescribe antibiotics to a patient diagnosed with viral infection with symptom level τ. Further, we have $\frac{\partial \Pi_P}{\partial \tau} = 0 \rightarrow \tau^*(w,b) = \arg\left\{0 \le \tau \le 1 : p(\tau) = \frac{q_2(\alpha F - k(\tau) + b)}{(1-q_1)(\alpha B(1-\delta) + k(\tau) - w - b) + q_2(\alpha F - k(\tau) + b)}\right\}$ and at this critical point, $\frac{\partial^2 \Pi_P}{\partial \tau^2}|_{\tau^*(w,b)} < 0 \Leftrightarrow w < \frac{k'(\tau^*)\left(q_2(2(1-p(\tau^*))p(\tau^*)(1-q_1)) + (p(\tau^*)(1-q_1))^2 + ((1-p(\tau^*))q_2)^2\right)}{p'(\tau^*(1-q_1)q_2} + \alpha(B - \delta B + F)$. Under such condition, if $b < \underline{b}^{\mathrm{T}}(0)$, then $\frac{\partial \Pi_P}{\partial \tau} < 0$, thus $\tau^*(w,b) = 0$. If $b > \underline{b}^{\mathrm{T}}(1)$, then $\frac{\partial \Pi_P}{\partial \tau} > 0$, thus $\tau^*(w,b) = 1$.

If $w > \frac{k'(\tau^*)\left(q_2(2(1-p(\tau^*))p(\tau^*)(1-q_1))+(p(\tau^*)(1-q_1))^2+((1-p(\tau^*))q_2)^2\right)}{p'(\tau^*)(1-q_1)q_2} + \alpha(B - \delta B + F)$, we have $\frac{\partial^2 \Pi_P}{\partial \tau^2} > 0$, which means $\tau^* = 0$ if $\Pi_P(\mathcal{P}(0), B, F, k(0), \zeta) > \Pi_P(\mathcal{P}(1), B, F, k(1), \zeta) \to b \leq \frac{(1-q_1)\left(\int_0^1 k(s)p(s)f_s(s)\,ds - \mathbb{E}(p)(w-\alpha B(1-\delta))\right)+q_2\left(\mathbb{E}(k)+\alpha F\mathbb{E}(p)-\alpha F-\int_0^1 k(s)p(s)f_s(s)\,ds\right)}{\mathbb{E}(p)(1-q_1)+(1-\mathbb{E}(p))q_2}$ and $\tau^* = 1$ otherwise. □

Proof of Proposition 4: Substituting Equation (9) in Equation (2) we have

$$\begin{aligned}
\Pi_S(\tau, B, F, c, h, \zeta) = &\left(\int_0^1 q_1 p(s) f_s(s)\,\mathrm{d}s + \int_\tau^1 (1-q_1)p(s)f_s(s)\,\mathrm{d}s\right)[U_S(B,F) - w - c] \\
&+ \left(\int_0^1 (1-q_2)(1-p(s))f_s(s)\,\mathrm{d}s + \int_\tau^1 q_2(1-p(s))f_s(s)\,\mathrm{d}s\right)[U_S(0,F) - w - c - h] \\
&+ \left(\int_0^\tau (1-q_1)p(s)f_s(s)\,\mathrm{d}s\right)[U_S(\delta B, F) - 2w - b - c] \\
&+ \left(\int_0^\tau q_2(1-p(s))f_s(s)\,\mathrm{d}s\right)[U_S(0,0) - w - b]
\end{aligned} \tag{A8}$$

From (A8), we have, $\frac{\partial \Pi_S}{\partial \tau} = \big(1-p(d)\big)q_2\big(F+c+h-b\big) - p(d)(1-q_1)\big(b + B\,(1-\delta)+w\big)f_s(\tau)$. Knowing the provider's best response, the payer would provide the minimum bonus $\underline{b}^{\mathrm{T}}(\tau)$ to prevent antibiotic prescription to a patient with symptom level τ if and only if $\frac{\partial \Pi_S}{\partial \tau} \geq 0 \Leftrightarrow \big(1-p(d)\big)q_2\big(F + c + h - \underline{b}^{\mathrm{T}}(\tau)\big) - p(d)(1-q_1)\big(\underline{b}^{\mathrm{T}}(\tau) + B\,(1-\delta)+w\big) \geq 0 \Leftrightarrow k(\tau) \leq \hat{k}^{\mathrm{T}}(\tau)$.

Solving OPT2, we have $\frac{\partial^2 \Pi_S}{\partial w^2} = 0$ and $\frac{\partial \Pi_S}{\partial w} = -1-(1-q_1)\int_0^\tau p(s)f_s(s)\,\mathrm{d}s < 0$. Therefore, regardless of the antibiotic prescription behaviour of the provider, the payer sets w such that it absorbs the surplus of the provider (binding individual-rationality constraint in OPT2). We know that, when $k(\tau) > \hat{k}^{\mathrm{T}}(\tau)$ the payer will not offer the minimum bonus required by the provider not to prescribe antibiotics to a patient with symptom level τ (i.e., $b < \underline{b}^{\mathrm{T}}(\tau)$). Therefore, we have the following.

1. When $k(0) > \hat{k}^{\mathrm{T}}(0) \to b < \underline{b}^{\mathrm{T}}(0)$, and thus $\tau^* = 0$. In this case, $\Pi_P|_{b<\underline{b}^{\mathrm{T}}(0);\tau^*=0} = \underline{\Pi}_P \Leftrightarrow w = \underline{\Pi}_P + \alpha(F - B\mathbb{E}(p))$.

2. When $\hat{k}^{\mathrm{T}}(1) \leq k(\tau) \leq \hat{k}^{\mathrm{T}}(0)$, since $\frac{\partial \hat{k}^{\mathrm{T}}(\tau)}{\partial \tau} < 0$, the payer will offer the minimum bonus required by the provider (i.e., $b = \underline{b}^{\mathrm{T}}(\tau^*)$) up to a patient with symptom level τ^* such that $k(\tau^*(w,b)) = \hat{k}^{\mathrm{T}}(\tau^*(w,b))$ which yields $b = \frac{k(\tau)(B(1-\delta)+c+F+h+w)-wF(1+\alpha)-(c+h)(w-\alpha B(1-\delta))}{(1+\alpha)F+c+h+(1+\alpha)B(1-\delta)}$. Replacing b in $\tau^*(w,b)$ we get $\tau^* = \arg\left\{0 \leq \tau \leq 1 : p(\tau) = \frac{q_2((1+\alpha)F+c+h-k(\tau))}{q_2((1+\alpha)F+c+h-k(\tau))+(1-q_1)((1+\alpha)B(1-\delta)+k(\tau))}\right\}$. In this case, $\Pi_P|_{b=\underline{b}(\tau^*);\tau^*} = \underline{\Pi}_P \Leftrightarrow w = \frac{\underline{\Pi}_P+\alpha(F-B\mathbb{E}(p))-\alpha(B(1-\delta)+F)\Phi}{1-\Phi}$ where $\Phi = \frac{q_2(c+(1+\alpha)F+h-k)(\int_0^{\tau^*} f_s(s)ds+\int_0^{\tau^*} p(s)f_s(s)ds)-(1-q_1)(k+B(1+\alpha)(1-\delta))\int_0^{\tau^*} p(s)f_s(s)ds}{c+(1+\alpha)F+h+B(1+\alpha)(1-\delta)}$.

3. In the paper, we assume that $\hat{k}^{\mathrm{T}}(1) \leq 0 \leq k(1)$; however, if this is not the case, when $k(1) < \hat{k}^{\mathrm{T}}(1) \to b > \underline{b}^{\mathrm{T}}(1)$, and thus $\tau^* = 1$. In this case, $\Pi_P|_{b=\underline{b}^{\mathrm{T}}(1);\tau^*=1} = \underline{\Pi}_P \Leftrightarrow w = \frac{q_2\left(\underline{b}^{\mathrm{T}}(1)(\mathbb{E}(p)-1)+\mathbb{E}(k)+\alpha(\mathbb{E}(p)-1)F-\int_0^1 k(s)p(s)f_s(s)\,ds\right)-\underline{b}^{\mathrm{T}}(1)\mathbb{E}(p)(1-q_1)+\alpha(F-B\mathbb{E}(p)(\delta(1-q_1)+q_1))+(1-q_1)\left(\int_0^1 k(s)p(s)f_s(s)\,ds\right)+\underline{\Pi}_P}{\mathbb{E}(p)(1-q_1)+1}$.

□

Proof of Corollary 2: Comparing the optimal prescription thresholds from cases where diagnosis relies on interpretation of symptom presentation (define $\tau^{\mathrm{P}*}$) to those where diagnosis depends on additional diagnostic testing (define $\tau^{\mathrm{T}*}$), we have $\frac{q_2((1+\alpha)F+c+h-k(\tau))}{q_2((1+\alpha)F+c+h-k(\tau))+(1-q_1)((1+\alpha)B(1-\delta)+k(\tau))} > \frac{(1+\alpha)F+c+h-k(\tau)}{(1+\alpha)F+c+h+(1+\alpha)B(1-\delta)} \Leftrightarrow k(\tau) < (1+\alpha)F+c+h$ (which is required to ensure non-negative prescription threshold) that is $p(\tau^{\mathrm{T}*}) > p(\tau^{\mathrm{P}*})$ and thus $\tau^{\mathrm{T}*} > \tau^{\mathrm{P}*}$ because $p(\tau)$ is increasing in τ. Therefore, comparing true viral patients who do not receive antibiotics when diagnosis relies on additional diagnostic testing to the case when diagnosis depends on interpretation of symptom presentation, we have $q_2\int_0^{\tau^{\mathrm{T}*}}(1-p(s))f_s(s)\,\mathrm{d}s \geq \int_0^{\tau^{\mathrm{P}*}}(1-p(s))f_s(s)\,\mathrm{d}s \Leftrightarrow q_2 \geq \frac{\int_0^{\tau^{\mathrm{P}*}}(1-p(s))f_s(s)\,\mathrm{d}}{\int_0^{\tau^{\mathrm{T}*}}(1-p(s))f_s(s)\,\mathrm{d}s} \equiv \underline{q_2}$. $\square$

Proof of Corollary 3: When the payer offers $\zeta=\{w\}$, we remove bonus b from (A7) and (A8) to get the provider and the payer's payoff functions, respectively. For the provider, we have,

$$\frac{\partial^2\Pi_P|_{\zeta=\{w\}}}{\partial\tau^2} = f_s'(\tau)\left((1-q_1)p(\tau)(w-\alpha B(1-\delta)-k(\tau))+q_2((1-p(\tau))(\alpha F-k(\tau)))\right)$$
$$+f_s(\tau)\left(q_2\left(p'(\tau)(k(\tau)-\alpha F)-(1-p(\tau))k'(\tau)\right)-(1-q_1)\left(p'(\tau)(k(\tau)-\alpha B\delta+\alpha B-w)+p(\tau)k'(\tau)\right)\right)$$

and $\frac{\partial\Pi_P|_{\zeta=\{w\}}}{\partial\tau} = ((1-q_1)p(\tau)(w-\alpha B(1-\delta)-k(\tau))+q_2((1-p(\tau))(\alpha F-k(\tau))))f_s(\tau)$. We can show that $\frac{\partial\Pi_P|_{\zeta=\{w\}}}{\partial\tau}<0 \Leftrightarrow w < \frac{p(1-q_1)(\alpha B(1-\delta)+k)+(1-p)q_2(k-\alpha F)}{p(1-q_1)} = \underline{w}^{\mathrm{T}}(\tau)$ – minimum visit fee required for the provider not to prescribe antibiotics to a patient with symptom level τ. Further, we have $\frac{\partial\Pi_P|_{\zeta=\{w\}}}{\partial\tau}=0 \rightarrow \tau^*(w)=\arg\left\{0\leq\tau\leq1 : p(\tau)=\frac{q_2(\alpha F-k(\tau))}{(1-q_1)(\alpha B(1-\delta)+k(\tau)-w)+q_2(\alpha F-k(\tau))}\right\}$ and at this critical point, $\frac{\partial^2\Pi_P|_{\zeta=\{w\}}}{\partial\tau^2}|_{\tau^*(w)}<0 \Leftrightarrow w < \frac{k'(\tau^*)\left(q_2(2(1-p(\tau^*))p(\tau^*)(1-q_1))+(p(\tau^*)(1-q_1))^2+((1-p(\tau^*))q_2)^2\right)}{p'(\tau^*(1-q_1)q_2} + \alpha(B-\delta B+F)$. Under such condition, if $w<\underline{w}^{\mathrm{T}}(0)$, then $\frac{\partial\Pi_P|_{\zeta=\{w\}}}{\partial\tau}<0$, thus $\tau^*(w)=0$. If $w>\underline{w}^{\mathrm{T}}(1)$, then $\frac{\partial\Pi_P|_{\zeta=\{w\}}}{\partial\tau}>0$, thus $\tau^*(w)=1$.

On the other hand, for the payer, we have $\frac{\partial\Pi_S|_{\zeta=\{w\}}}{\partial\tau} = \big(q_2(1-p(\tau))(c+F+h)-(1-q_1)p(\tau)(B(1-\delta)+w)\big)f_s(\tau)$. Knowing the provider's best response, the payer *may* provide the minimum visit $\underline{w}^{\mathrm{T}}(\tau)$ to prevent antibiotic prescription to a viral-diagnosed patient with symptom level τ if and only if $\frac{\partial\Pi_S|_{\zeta=\{w\}}}{\partial\tau}\geq0 \Leftrightarrow \big(q_2(1-p(\tau))(c+F+h)-(1-q_1)p(\tau)(B(1-\delta)+\underline{w}^{\mathrm{T}}(\tau))\big)f_s(\tau)\geq0 \Leftrightarrow k(\tau)\leq\hat{k}^{\mathrm{T}}(\tau)$.

However, unlike in the $\zeta=\{w,b\}$ contract, with $\zeta=\{w\}$ the payer may not always want to disincentivize antibiotic prescription when $k(\tau)\leq\hat{k}^{\mathrm{T}}(\tau)$). This is because, with $\zeta=\{w\}$, the payer cannot absorb the provider's surplus and achieve optimal antibiotic prescription behaviour simultaneously. We have $\frac{\partial^2\Pi_S|_{\zeta=\{w\}}}{\partial w^2}=0$ and $\frac{\partial\Pi_S|_{\zeta=\{w\}}}{\partial w}<0$; therefore, instead of disincentivizing antibiotic prescription, the payer can offer the lowest possible visit fee by binding the individual rationality constraint in OPT2: $\Pi_P(\tau(w))=\underline{\Pi}_P \Leftrightarrow w = \frac{\underline{\Pi}_P+\alpha(B(1-\delta)(1-q_1)+Fq_2)\int_0^\tau p(s)f_s(s)\,ds-\alpha B\mathbb{E}(p)+\alpha F-\alpha Fq_2\int_0^\tau f_s(s)\,dy-(q_1+q_2-1)\int_0^\tau k(s)p(s)f_s(s)\,ds+q_2\int_0^\tau k(s)f_s(s)\,ds}{1+(1-q_1)\int_0^\tau p(s)f_s(s)ds} = \underline{\underline{w}}^{\mathrm{T}}$. When $k(\tau)\leq\hat{k}^{\mathrm{T}}(\tau)$ we have: $\underline{\underline{w}}^{\mathrm{T}}\neq\underline{w}^{\mathrm{T}}$. We can show that $\exists B,F,c,h\in\mathbb{R}_{\geq0} : \Pi_S|_{w=\underline{w}^{\mathrm{T}};\tau^*}\leq$

$\Pi_S|_{w=\underline{\underline{w}}^{\mathrm{T}};\tau}$. Therefore, with $\zeta = \{w\}$ the payer does not prefer to achieve the socially optimal antibiotic prescription levels. □

E-Companion B – Case Study: Sore throat presenting to primary care

B.1 Background

Sore throat represents 3-6% of all family physician office visits (Worrall et al. 2007). In adults, 85-90% of all sore throats are caused by viral infections and so antibiotics are not indicated (Worrall et al. 2007, Linder and Stafford 2001). The main bacterial cause of sore throat is group A β-hemolytic streptococci (GABHS or 'strep throat'). GABHS is the only common cause of sore throat warranting antibiotic treatment (Linder and Stafford 2001). Left untreated, GABHS infection can result in serious complications. Antibiotics provide symptom relief, reduce rates of suppurative peritonsillar and retropharyngeal abscess, acute rheumatic fever, and poststreptococcal glomerulonephritis (Kalra et al. 2016, Choby 2009) Antibiotics have side effects experienced by patients who take them, but also contribute to the social harm of antibiotic resistance. Despite these harms, inappropriate use of antibiotics for the treatment of sore throat is widespread, with antibiotics prescribed in over 70% of visits (Linder and Stafford 2001).

We estimate the value of antibiotics in a patient with bacterial infection from averting long-term sequelae of infection, B, and the consequences of adverse events associated with antibiotic use F. Health benefits and harms include health system costs, as well as changes in life expectancy, and quality-of-life. For this analysis, changes in life expectancy and quality-of-life were valued at $50,000 per quality-adjusted life-year (QALY)-gained, consistent with the marginal value of QALYs used in analysis by the Canadian Agency for Drugs and Technologies in Health (CADTH). When necessary, we converted to Canadian currency and adjusted for inflation using the Consumer Price Index presenting all costs in 2019 Canadian dollars. Base case parameter values are presented in Table 1.

B.2 Parameterization and Assumptions

Probability of bacterial infection (p) Individual symptoms that commonly occur concomitantly with sore throat each individually have relatively low sensitivity and specificity for diagnosing GABHS infection (Ebell et al. 2000). The modified Centor score and FeverPAIN scale categorize patients into risk groups based on symptom presentation (Choby 2009, Kalra et al. 2016). For example, patients with a modified Centor score of 0 have a 1-2.5% risk of GABHS infection, a score of 1 have a 5-10% risk of GABHS infection, a score of 2 have a 11-17% risk of GABHS infection, and so on (Choby 2009). Evidence-based guidelines for patients presenting with an intermediate score indicates the use of throat culture or rapid antigen test to assist in diagnosis (Choby 2009). For the purpose of this case study we focus on patients with an low and intermediate risk of GABHS infection using a base case value of 10%.

Table 1 Parameter inputs for numerical analysis

Model parameter	Base case value	Range	Reference
Prevalence of bacterial infection (p)			
Proportion of sore throat patients with bacterial infection	10%	0-50%	Choby (2009)
Test accuracy (q_1 and q_2)			
Without rapid test			
Sensitivity	75.0%	68 - 82%	Humair et al. (2006), McIsaac et al. (2004)
Specificity	48.7%	42 - 55%	Humair et al. (2006), McIsaac et al. (2004)
With rapid test			
Sensitivity	91.4%	87 - 96%	Humair et al. (2006)
Specificity	95.3%	92 - 98%	Humair et al. (2006)
Benefits of antibiotics in a patient with bacterial infection (B)			
Symptom improvement (16 hours)	$69.86	$58.90-69.86	See text
Peritonsillar abscess			
Absolute reduction in risk	0.022%	0.01-0.035%	Spinks et al. (2021)
Cost	$2579	$2236-3268	Canadian Institute for Health Information (CIHI) (Accessed: April 11, 2023)
Rhuematic fever			
Absolute reduction in risk	0.09%	0.02-0.35%	Spinks et al. (2021), Cooper et al. (2001)
Cost	$239,830	$107,105-372,263	See text
Poststreptococcal glomerulonephritis			
Absolute reduction in risk	0.09%	0.02-0.35%	Spinks et al. (2021)
Cost	$10,588	$7,595-17,679	Canadian Institute for Health Information (CIHI) (Accessed: April 11, 2023)
Total	$357.486		Calculated
Harms of antibiotic use (F)			
Adverse events	$2.96	$2.50-3.55	See text
Societal harm of antibiotic use from resistance (h)			
Per-prescription cost of resistance	$80	$0-220	See text
Cost of antibiotics (c)			
Per-prescription cost of amoxicillin	$12.55		Ontario Ministry of Health and Long Term Care (2023a,b)
Other assumptions			
Physician altruism factor (α)	0.2		Assumption
Reduced benefit from delayed treatment (δ)	0.86		Assumption
Per-visit reimbursement ($\underline{\Pi}_P$)	37.95		Ontario Ministry of Health and Long Term Care (2023c)
Opportunity cost (k)	37.95		Assumption

Diagnostic accuracy There are several clinical rules that can aid physicians attempting to differentiate viral and bacterial causes of sore throat (e.g., Centor score and FeverPAIN score). Using these scoring systems, empirical antibiotic treatment is recommended for patients with very high scores representing more quintessential symptoms and risk factors for bacterial infection; no antibiotic treatment or additional testing is recommended for patients with low scores; and, additional testing is recommended for patients with intermediate scores (Kalra et al. 2016). Studies in which these tools were applied identified very high rates of antibiotic prescription using clinical rules alone. Classification of bacterial infections based on clinical presentation alone, assisted by the modified Centor score or the FeverPAIN scale, has a sensitivity of 75.0% and specificity of 48.7% (Humair et al. 2006, McIsaac et al. 2004). Systematic use of rapid antigen tests indicate improved diagnostic accuracy and reduced inappropriate antibiotic prescribing. Rapid antigen tests improve diagnostic classification of bacterial infections with a sensitivity of 91.4% and specificity of 95.3% (Humair et al. 2006).

Benefits of antibiotics in patients with GABHS (B) The benefits of antibiotics in patients with GABHS are symptom relief and reductions in the possible consequences of an untreated bacterial infection such as suppurative peritonsillar abscess, rheumatic fever, and poststeptococcal glomerulonephritis (Choby 2009, Kalra et al. 2016).

Symptom Relief: Patients who test positive for GABHS infection who do receive penicillin experience symptom relief about 16 hours earlier than those who test negative (Worrall et al. 2007). We estimated the health benefit of one-day of symptom relief assuming the quality-of-life reduction associated with symptoms was 0.51 based on a study of quality-of-life in patients with outpatient influenza (Hollmann et al. 2013). We then converted to a monetary health benefit using the willingness to pay threshold of $50,000 per quality-adjusted life-year (QALY)-gained.

Suppurative peritonsillar abscess: Suppurative peritonsillar abscess is a local collection of pus in the peritonsillar spaces (loose connective tissues at back of the throat) (Gupta and McDowell 2022). Antibiotics reduce the risk of this complication from 2.4% to 0.14% of GABHS infections (Spinks et al. 2021). Treatment of peritonsillar abscess requires surgical drainage and antibiotics. Caught early, this complication can be treated in the emergency department and does not require overnight hospital stay. In rare cases, infection can occur in the retropharyngeal space, threatening the airway, and requiring more complicated surgical intervention and inpatient care. As an estimate of average treatment cost, we used the average Canadian cost of adult hospitalization for Tonsillitis/Pharyngitis in 2019 of $2359. We assume that the quality of life reduction associated

with peritonsillar abscess lasts 7 days and, based on an empiric study in patients, that the daily quality-of-life reduction associated with peritonsillar abscess is 0.23 (Hackenberg et al. 2022).

Rheumatic fever: Rheumatic fever is a condition that can inflame the heart, joints, brain, and skin typically occurring 1-5 weeks after a GABHS infection (de Loizaga and Beaton 2021). Approximately 60% of patients with rheumatic fever will develop chronic rheumatic heart disease (de Loizaga and Beaton 2021). Studies conducted in the 1950s and 1960s indicate a 2-3% risk of rheumatic fever in the 2 months following untreated GABHS infection, a risk reduced by 70-80% with antibiotic treatment (Spinks et al. 2021, Robertson et al. 2005). Although there remain serious reports of ongoing risk of rheumatic fever in marginalized populations (Gordon et al. 2015, Lennon et al. 2009), the overall incidence of acute rheumatic fever was 60-times greater in 1965 than in 1994, in part, due to wide-spread use of antibiotics for prevention (Cooper et al. 2001). Therefore, we estimate antibiotics reduces the risk of rheumatic fever from 0.12% to 0.03% in the general population.

We estimated the initial cost of rheumatic fever based on the average Canadian cost of hospitalization for fever (Canadian Institute for Health Information (CIHI) Accessed: April 11, 2023). We then estimated the cost of screening, diagnosis, and treatment of rheumatic heart disease using a cost-effectiveness analysis of screening for rheumatic heart disease Roberts et al. (2017). This study did not have a lifetime analysis horizon, so we further inflated costs based on another study of rheumatic heart disease that found two-thirds of the mean lifetime cost occurs after the age of 30 (Milne et al. 2012).

Poststeptococcal glomerulonephritis: Poststeptococcal glomerulonephritis involves the rapid deterioration of kidney function associated with an inflammatory response following a GABHS infection. Very rarely, poststeptococcal glomerulonephritis can lead to death or chronic kidney failure. Because it is such a rare complication, most studies of antibiotics for sore throat identify no events in either study arm (Spinks et al. 2021). Based on a Cochrane review, we estimate antibiotics reduces the risk of poststeptococcal glomerulonephritis from 0.09% to 0.00% (Spinks et al. 2021). We estimate the cost of treatment based on the CIHI Canadian average cost of hospitalization for kidney disease in 2019 ($9581) and the monetary value of a 0.35 quality-of-life lost for 3-weeks consistent with a quality of life study in patients with chronic kidney disease (Canadian Institute for Health Information (CIHI) Accessed: April 11, 2023, Cooper et al. 2020).

Harms of antibiotics in patients with sore throat (F) We estimated the rates of common or severe adverse events associated with antibiotic treatment of sore throat using large population based studies and meta-analyses (Thornhill et al. 2015, Soukavong et al. 2016, Gillies et al.

2015). Based on a 51-year study of amoxicillin use, we estimated the rate of fatal adverse event to be 0.11 per million prescriptions (Thornhill et al. 2015). We estimated the rates of non-fatal adverse events as follows: Rash (11.4%), Hives (6.6%), Diarrhea (5.8%), Dry and itchy skin (4.9%), Nausea/Vomiting (4.6%), Yeast infection (4.4%), Fever (2.3%), Swelling (2.0%), and Indigestion (1.8%) (Soukavong et al. 2016, Gillies et al. 2015). Consistent with the analysis in other sections, we valued premature death at $50,000 per QALY resulting in an estimated value of $1,195,709 for an average adult. In sensitivity analysis, we used $6.5 million as the statistical value of life in Canada (Chestnut and De Civita 2009). For non-fatal adverse events, we estimated the cost of care to be that of 5-days of appropriate over-the-counter treatments based on current pharmacy prices.

Societal consequences of antibiotic resistance, (h) The US Centers for Disease Control and Prevention conservatively estimates the current annual health care cost of antimicrobial resistance in the US to be $20 billion (Dadgostar 2019). Dividing by the annual number of outpatient antibiotic prescriptions in the US (251.1 million), we estimate the societal harm of antibiotic resistance per prescription to be $80.

Cost of antibiotics (c) Penicillin, specifically amoxicillin, is the recommended antibiotic for treatment of GABHS infection because of its narrow spectrum of activity, cost, few adverse effects, and effectiveness (Kalra et al. 2016). The cost of a 10-day course of amoxicillin (500 mg twice daily) through the Ontario Drug Benefit plan is $2.62 ($0.131 per 500 mg capsule) (Ontario Ministry of Health and Long Term Care 2023a). Including the $9.93 pharmacy dispensing fee, the total cost is $12.55. (Ontario Ministry of Health and Long Term Care 2023b)

Other assumptions We estimate $\underline{\Pi}_P$, which conceptually includes both the payment fee and the utility the provider receives from patient well-being, using the current current per-visit fee for an intermediate assessment by a family doctor of $37.95 (Ontario Ministry of Health and Long Term Care 2023c). The opportunity cost for providers who explain to patients that antibiotics are not indicated for their viral diagnosis includes the time it takes to explain, as well as the concern the provider has about the risk of misdiagnosis, patient disappointment/satisfaction, and risking a positive relationship with the patient (Butler et al. 1998, Dempsey et al. 2014, Silverman et al. 2017). Because higher rates of overprescribing are so strongly associated with greater patient throughput, we use $37.95, the full per-visit fee, to estimate the opportunity cost k. Because of differences in physician response to the perceived expectation of patients for antibiotics and challenges communicating with patients due to age, education, and provider's relationship with

their patients (Dempsey et al. 2014, Kitano et al. 2021), there is heterogeneity in the expected opportunity cost of providers. We explore the effect of this heterogeneity in sensitivity analysis.

To complete the numerical analysis, we needed to select values for the multiplier used to reduce clinical benefit from delayed treatment (δ) and the multiplier used to represent provider altruism as a proportion of the payer's value for patient outcomes (α). In developed economies, sequelae from untreated or delayed treatment of GABHS is extremely rare, but there is no specific estimate for the consequences of a 1-2 week delay in treatment for otherwise healthy adults. Consistent with the strong recommendation to not provide antibiotics to people diagnosed with a viral infection, we selected the value of δ to be the lowest value for which that recommendation was still clinically rational ($\delta = 0.86$). When provider altruism (α) is zero, providers are concerned exclusively with their own compensation and place no value on the quality of their own work. When provider altruism is one, providers place the same value on patient outcomes as the payer, placing value on the patient's outcomes over their own. We choose $\alpha = 0.2$ for our analysis which results in similarly scaled values for the utility associated with patient well-being and provider income in patients with bacterial infections. We do not include a cost of the rapid antigen diagnostic test which costs \$5.70 including physician administration of the test (Ontario Ministry of Health and Long Term Care 2023c).

Finally, to illustrate results, we need to assume a distribution for s and a function for the probability of bacterial infection conditional on symptom level s. For simplicity, we assume patients present with a uniform distribution over possible symptoms, i.e., $f_S(s) = 1$, and the probability of bacterial infection is linearly increasing in s, i.e., $p(s) = 2\mathbb{E}(p)s$.

References

Butler CC, Rollnick S, et al. (1998) Understanding the culture of prescribing: qualitative study of general practitioners' and patients' perceptions of antibiotics for sore throats. *BMJ* 317(7159):637–642.

Canadian Institute for Health Information (CIHI) (Accessed: April 11, 2023) Patient cost estimator. URL https://www.cihi.ca/en/patient-cost-estimator.

Chestnut L, De Civita P (2009) Economic valuation of mortality risk reduction. *Ottawa (ON): Government of Canada, Policy Research Initiative* 1–69.

Choby BA (2009) Diagnosis and treatment of streptococcal pharyngitis. *Am Fam Physician* 79(5):383–390.

Cooper JT, Lloyd A, Sanchez JJG, Sörstadius E, Briggs A, McFarlane P (2020) Health related quality of life utility weights for economic evaluation through different stages of chronic kidney disease: a systematic literature review. *Health Qual Life Outcomes* 18:1–11.

Cooper RJ, Hoffman JR, Bartlett JG, Besser RE, Gonzales R, Hickner JM, Sande MA (2001) Principles of appropriate antibiotic use for acute pharyngitis in adults: background. *Ann Intern Med* 134(6):509–517.

Dadgostar P (2019) Antimicrobial resistance: implications and costs. *Infect Drug Resist* 3903–3910.

de Loizaga SR, Beaton AZ (2021) Rheumatic fever and rheumatic heart disease in the United States. *Pediatr Ann* 50(3):e98–e104.

Dempsey PP, Businger AC, Whaley LE, Gagne JJ, Linder JA (2014) Primary care clinicians' perceptions about antibiotic prescribing for acute bronchitis: a qualitative study. *BMC Fam Pract* 15(1):1–10.

Ebell MH, Smith MA, Barry HC, Ives K, Carey M (2000) Does this patient have strep throat? *JAMA* 284(22):2912–2918.

Gillies M, Ranakusuma A, Hoffmann T, Thorning S, McGuire T, Glasziou P, Del Mar C (2015) Common harms from amoxicillin: a systematic review and meta-analysis of randomized placebo-controlled trials for any indication. *CMAJ* 187(1):E21–E31.

Gordon J, Kirlew M, Schreiber Y, Saginur R, Bocking N, Blakelock B, Haavaldsrud M, Kennedy C, Farrell T, Douglas L, et al. (2015) Acute rheumatic fever in first nations communities in northwestern ontario: Social determinants of health "bite the heart". *Can Fam Physician* 61(10):881–886.

Gupta G, McDowell RH (2022) Peritonsillar abscess. *StatPearls [Internet] Treasure Island (FL): StatPearls Publishing; PMID: 30137805* .

Hackenberg B, Büttner M, Schöndorf M, Strieth S, Schramm W, Matthias C, Gouveris H (2022) Quality of life assessment for tonsillar infections and their treatment. *Medicina* 58(5):589.

Hollmann M, Garin O, Galante M, Ferrer M, Dominguez A, Alonso J (2013) Impact of influenza on health-related quality of life among confirmed (h1n1) 2009 patients. *PloS one* 8(3):e60477.

Humair JP, Revaz SA, Bovier P, Stalder H (2006) Management of acute pharyngitis in adults: reliability of rapid streptococcal tests and clinical findings. *Arch Intern Med* 166(6):640–644.

Kalra MG, Higgins KE, Perez ED (2016) Common questions about streptococcal pharyngitis. *Am Fam Physician* 94(1):24–31.

Kitano T, Langford BJ, et al. (2021) The association between high and unnecessary antibiotic prescribing: a cohort study using family physician electronic medical records. *Clin Infect Dis* 72(9):e345–e351.

Lennon D, Stewart J, Farrell E, Palmer A, Mason H (2009) School-based prevention of acute rheumatic fever: a group randomized trial in new zealand. *Pediatr Infect Dis J* 28(9):787–794.

Linder JA, Stafford RS (2001) Antibiotic treatment of adults with sore throat by community primary care physicians: a national survey, 1989-1999. *JAMA* 286(10):1181–1186.

McIsaac WJ, Kellner JD, Aufricht P, Vanjaka A, Low DE (2004) Empirical validation of guidelines for the management of pharyngitis in children and adults. *JAMA* 291(13):1587–1595.

Milne RJ, Lennon D, Stewart JM, Vander Hoorn S, Scuffham PA (2012) Mortality and hospitalisation costs of rheumatic fever and rheumatic heart disease in New Zealand. *J Paediatr Child Health* 48(8):692–697.

Ontario Ministry of Health and Long Term Care (2023a) Ontario drug benefit (2023). URL https://www.formulary.health.gov.on.ca/formulary/results.xhtml?q=amoxicillin&type=1.

Ontario Ministry of Health and Long Term Care (2023b) Ontario drug benefit program: Dispensing fees. URL https://www.health.gov.on.ca/en/public/programs/drugs/programs/odb/opdp_dispensing_fees.aspx.

Ontario Ministry of Health and Long Term Care (2023c) Schedule of benefits: Physician services under the health insurance act (March 9, 2023 (effective April 1, 2023)). URL https://www.health.gov.on.ca/en/pro/programs/ohip/sob/.

Roberts K, Cannon J, Atkinson D, Brown A, Maguire G, Remenyi B, Wheaton G, Geelhoed E, Carapetis JR (2017) Echocardiographic screening for rheumatic heart disease in indigenous australian children: A cost–utility analysis. *J Am Heart Assoc* 6(3):e004515.

Robertson KA, Volmink JA, Mayosi BM (2005) Antibiotics for the primary prevention of acute rheumatic fever: a meta-analysis. *BMC Cardiovasc Disord* 5(1):1–9.

Silverman M, Povitz M, Sontrop JM, Li L, Richard L, Cejic S, Shariff SZ (2017) Antibiotic prescribing for nonbacterial acute upper respiratory infections in elderly persons. *Ann Intern Med* 166(11):765–774.

Soukavong M, Kim J, Park K, Yang BR, Lee J, Jin XM, Park BJ (2016) Signal detection of adverse drug reaction of amoxicillin using the Korea adverse event reporting system database. *Journal of Korean Medical Science* 31(9):1355–1361.

Spinks A, Glasziou PP, Del Mar CB (2021) Antibiotics for treatment of sore throat in children and adults. *Cochrane Database Syst Rev* (12).

Thornhill MH, Dayer MJ, Prendergast B, Baddour LM, Jones S, Lockhart PB (2015) Incidence and nature of adverse reactions to antibiotics used as endocarditis prophylaxis. *J Antimicrob Chemother* 70(8):2382–2388.

Worrall G, Hutchinson J, Sherman G, Griffiths J (2007) Diagnosing streptococcal sore throat in adults: randomized controlled trial of in-office aids. *Can Fam Physician* 53(4):666–671.

E-Companion C – Extensions

C1: Bonus and second visit fee for Outcome C

In this section, we present the analysis for the case when there is no second-visit fee and bonus payment for patients with health Outcome C. Modifying the payments for Outcome C, the provider's expected payoff function (Equation (1) in the paper) will change to

$$\Pi_P(\tau,B,F,k,\zeta)=\int_0^1\begin{pmatrix} P_A(s,\tau)\left[U_P(B,F)+w\right]+P_B(s,\tau)[U_P(0,F)+w] \\ +P_C(s,\tau)[U_P(\delta B,F)+w-k(s)]+P_D(s,\tau)[U_P(0,0)+w+b-k(s)]\end{pmatrix} f_s(s)\,\mathrm{d}s, \tag{E1}$$

Further, the payer's expected payoff function (Equation (2) in the paper) takes the form of

$$\Pi_S(\tau,B,F,c,h,\zeta)=\int_0^1\begin{pmatrix} P_A(s,\tau)\left[U_S(B,F)-w-c\right]+P_B(s,\tau)[U_S(0,F)-w-c-h] \\ +P_C(s,\tau)[U_S(\delta B,F)-w-c]+P_D(s,\tau)[U_S(0,0)-w-b]\end{pmatrix} f_s(s)\,\mathrm{d}s. \tag{E2}$$

Despite the changes to the provider and the payer's expected payoff functions (because of the changes to the payments for health Outcome C), there will not be any changes to the central planner's objective function (Equation (4) in the paper) when we remove the follow-up visit fee and bonus payment from Outcome C. This is because the central planner does not consider the transfer payments between the payer and the provider when solving for the socially optimal levels of antibiotic prescription. Hence, the socially optimal levels of antibiotic prescription (Proposition 1 and 3) will not be affected by the changes to the contract payments.

When diagnosis relies on symptom presentation: In this case the the minimum bonus required for the provider to not prescribe antibiotics to a patient with symptom level τ or lower is $\underline{b}^{\mathrm{P}}(\tau)$, where

$$\underline{b}^{\mathrm{P}}(\tau)=k(\tau)-\alpha F+p(\tau)\left(\alpha\left(B-\delta B+F\right)+b\right). \tag{E3}$$

This minimum required bonus payment (Equation (E3)) is larger than the one in main model (Equation (6) in the paper). Given the altered minimum required bonus payment, the provider's optimal prescription threshold (cf. Lemma 2) will change to a threshold such that at $\tau^*(b)$ we have $p(\tau)=\frac{\alpha F-k(\tau)+b}{\alpha(B-\delta B+F)+b}$ that results in a smaller prescription threshold compared to the main model. This is because without the follow-up visit fee and bonus payment for Outcome C the provider does not have incentive to misdiagnose patients on purpose to increase visit payments.

We have, $\frac{\partial\Pi_S}{\partial\tau}=(p(\tau)(-(B-\delta B)+b)+(1-p(\tau))(F+c+h)-b)\,f_s(\tau)$. Thus, $\frac{\partial\Pi_S}{\partial\tau}\geq 0\Leftrightarrow b\leq c+F+h-\frac{(B(1-\delta))p(\tau)}{1-p(\tau)}$. Knowing the provider's best response, the payer would provide the minimum bonus $\underline{b}^{\mathrm{P}}(\tau)$ to prevent antibiotic prescription to a patient with symptom level τ if and only if $\underline{b}^{\mathrm{P}}(\tau)\leq c+F+h-\frac{(B(1-\delta))p(\tau)}{1-p(\tau)}\Leftrightarrow k(\tau)\leq\hat{k}^{\mathrm{P}}(\tau)$. Similar to the main model when $k(\tau)>\hat{k}^{\mathrm{P}}(\tau)$ the

payer will not offer the minimum bonus required by the provider not to prescribe to a patient with symptom level τ (i.e., $b < \underline{b}^{\mathrm{P}}(\tau)$). Therefore, we have the following.

1. When $k(0) > \hat{k}^{\mathrm{P}}(0) \to b < \underline{b}^{\mathrm{P}}(0)$, and thus $\tau^* = 0$. In this case, $\Pi_P|_{b<\underline{b}^{\mathrm{P}}(0);\tau^*=0} = \underline{\Pi}_P \Leftrightarrow w = \underline{\Pi}_P + \alpha(F - B\mathbb{E}(p))$. Therefore, there is no change to the first part of Proposition 2 from the main model.

2. When $k(0) \le \hat{k}^{\mathrm{P}}(0)$, since $\frac{\partial \hat{k}^{\mathrm{P}}(\tau)}{\partial \tau} < 0$, the payer will offer the minimum bonus required by the provider (i.e., $b = \underline{b}^{\mathrm{P}}(\tau^*)$) up to a patient with symptom level τ^* such that $k = \hat{k}^{\mathrm{P}}(\tau^*(b))$ which yields $b = \frac{B(1-\delta)(\alpha(c+h)+k(\tau))+k(\tau)(c+F+h)}{(\alpha+1)B(1-\delta)+k(\tau)}$. Replacing b in $\tau^*(b)$ we get $\tau^* = \arg\left\{0 \le \tau \le 1 : p(\tau) = \frac{(1+\alpha)F+c+h-k(\tau)}{(1+\alpha)F+c+h+(1+\alpha)B(1-\delta)}\right\}$ – that is the same antibiotic prescription behaviour as in the main model. In this case, w solves $\Pi_P|_{b=\underline{b}^{\mathrm{P}}(\tau^*);\tau^*} = \underline{\Pi}_P$.

When diagnosis relies on additional testing: Substituting Equation (9) in Equation (E1) we can show that for a provider with opportunity cost $k(\tau)$, the minimum reward payment, at a given visit fee w, that the provider requires not to prescribe antibiotics to a patient diagnosed with viral infection with symptom level τ is equal to $\underline{b}^{\mathrm{T}}(\tau)$, where

$$\frac{\partial \Pi_P}{\partial \tau} < 0 \Leftrightarrow b < \frac{-(1-p(\tau))q_2(\alpha F - k(\tau)) + p(\tau)(1-q_1)\left(\alpha B\left(1-\delta\right) + k(\tau)\right)}{(1-p(\tau))q_2} = \underline{b}^{\mathrm{T}}(\tau). \tag{E4}$$

Further, we have $\frac{\partial \Pi_P}{\partial \tau} = 0 \to \tau^*(b) = \arg\left\{0 \le \tau \le 1 : p(\tau) = \frac{q_2(\alpha F - k(\tau) + b)}{(1-q_1)(\alpha B(1-\delta)+k(\tau)) + q_2(\alpha F - k(\tau)+b)}\right\}$ and at this critical point, $\frac{\partial^2 \Pi_P}{\partial \tau^2}|_{\tau^*(b)} < 0$. Therefore, the provider will not prescribe antibiotics to patients diagnosed with viral infection with symptom level higher than $\tau^*(b)$ if and only if $b \ge \underline{b}^{\mathrm{T}}(\tau)$ (cf. Lemma 3). While Lemma 3 remains valid with the new payment structure, the minimum bonus payment required for not prescribing antibiotics to a viral diagnosis ($\underline{b}^{\mathrm{T}}(\tau)$) is not a function of the (re-)visit fee. Further, it follows from Equation (E2) that the payer balances their cost of over- and under-prescribing by providing the minimum bonus $\underline{b}^{\mathrm{T}}(\tau)$ to prevent antibiotic prescription to patients diagnosed with viral infection with symptom level τ if and only if

$$c + F + h + \frac{p(\tau)(1-q_1)(-(1-\delta)B)}{(1-p(\tau))q_2} - \underline{b}^{\mathrm{T}}(\tau) \ge 0. \tag{E5}$$

Substituting $\underline{b}^{\mathrm{T}}(\tau)$ from Equation (E4) into Equation (E5), we observe that, independent of visit fee w, the payer provides incentives when $k(0) \le \hat{k}^{\mathrm{T}}(0)$ and we get $\tau^* = \arg\left\{0 \le \tau \le 1 : p(\tau) = \frac{q_2((1+\alpha)F+c+h-k(\tau))}{q_2((1+\alpha)F+c+h-k(\tau)) + (1-q_1)((1+\alpha)B(1-\delta)+k(\tau))}\right\}$. This is the same condition under which antibiotics are not prescribed to viral-diagnosed patients with symptom level above τ in an integrated care system (Proposition 3). Therefore, an action-based payment contract will result in socially optimal levels of antibiotic prescription that is the same with or without the follow-up visit fee and bonus payment for Outcome C.

C2: When physicians care about the social harm of antibiotics

Below we present the analysis for the case when physicians also care about the social harm of antibiotics. Doing this modification on the model the objective function of the central planner (Equation (4) in the paper) will change to

$$\Pi_C(\tau, B, F, c, h, k) = \int_0^1 \begin{pmatrix} P_A(s,\tau)\,[U_S(B,F)+U_P(B,F)-c] + P_B(s,\tau)[U_S(0,F)+U_P(0,F)-c-(1+\gamma)h] \\ +P_C(s,\tau)[U_S(\delta B,F)+U_P(\delta B,F)-c-k(s)] + P_D(s,\tau)[U_S(0,0)+U_P(0,0)-k(s)] \end{pmatrix} f_s(s)\,\mathrm{d}s \tag{E6}$$

where $\gamma \in [0,1]$ is the degree of provider's concern about the social harm of antibiotic resistance. Therefore, consideration of the social harm by the two parties results in a higher impact of h on central planner's objective function.

When diagnosis relies on symptom presentation: When the provider cares about the social harm of antibiotic prescription, the opportunity cost threshold $\hat{k}^{\mathrm{P}}(\tau)$ is increasing as γ increases and this threshold is always larger than the the original model's opportunity cost threshold.

$$\hat{k}^{\mathrm{P}}(\tau) = (1+\alpha)F + c + (1+\gamma)h - p(\tau)\Big((1+\alpha)\,(B\,(1-\delta)+F) + c + (1+\gamma)h\Big) \tag{E7}$$

In this case, the socially optimal prescription threshold will change to $\tau^* = \arg\left\{0 \le \tau \le 1 : p(\tau) = \frac{(1+\alpha)F+c+(1+\gamma)h-k(\tau)}{(1+\alpha)F+c+(1+\gamma)h+(1+\alpha)B(1-\delta)}\right\}$ that is larger than the prescription threshold in Proposition 1. This means that fewer patients will be prescribed antibiotics when provider is concerned about the social harm of antibiotics. Further, the minimum acceptable bonus for the provider will also change such that the provider would require lower bonus to not prescribe antibiotics to patients with viral diagnosis. In particular, the $\underline{b}^{\mathrm{P}}(\tau)$ is smaller by $\gamma h(1-p(\tau))$ when we incorporate the social harm into the provider's objective function. This means that the provider incurs $\gamma h(1-p(\tau))$ higher cost of over-prescribing for a patient with symptom level τ. Despite the changes in the payments we show that the action-based payment contract can coordinate the system and achieve the socially optimal levels of antibiotic prescription.

When diagnosis relies on additional testing: Similarly, when additional tests are ordered for diagnosis, we show that $\hat{k}^{\mathrm{T}}(\tau)$ is larger than the main model when the provider cares about the social harm of antibiotic prescription. This means that fewer patients will be prescribed antibiotics when provider is concerned about the social harm of antibiotics. Further, the minimum acceptable bonus ($\underline{b}^{\mathrm{T}}(\tau)$) by the provider will also change such that the provider would require lower bonus to not prescribe antibiotics to patients diagnosed with viral infection. However, we show that the action-based payment contract can coordinate the system and achieve the socially optimal levels of antibiotic prescription. Note that the payments presented in Proposition 4 will be affected with this

change. However, we confirm that all of the structural results and insights presented in the paper are robust with respect to incorporating the social harm of antibiotics into the reward function of providers. □
